# Three randomized controlled trials evaluating the impact of “spin” in health news stories reporting studies of pharmacologic treatments on patients’/caregivers’ interpretation of treatment benefit

**DOI:** 10.1186/s12916-019-1330-9

**Published:** 2019-06-04

**Authors:** Isabelle Boutron, Romana Haneef, Amélie Yavchitz, Gabriel Baron, John Novack, Ivan Oransky, Gary Schwitzer, Philippe Ravaud

**Affiliations:** 1 INSERM, UMR 1153, Epidemiology and Biostatistics Research Center (CRESS), Methods Team, Paris, France; 2 Faculté de Médecine, Paris Descartes University, Paris, France; 3 Centre d’Épidémiologie Clinique, AP-HP (Assistance Publique des Hôpitaux de Paris), Hôpital Hôtel Dieu, 1, Place du parvis Notre Dame, 75004 Paris Cedex 4, France; 4 Inspire, Arlington, VA USA; 5 New York University’s Arthur Carter Journalism Institute, New York, USA; 6 HealthNewsReview.org, University of Minnesota, School of Public Health, Minneapolis, MN USA; 7 Department of Epidemiology, Columbia University Mailman School of Public Health, New York, NY USA

**Keywords:** Randomized trial, Spin, Distorted interpretation, Detrimental research practices

## Abstract

**Background:**

News stories represent an important source of information. We aimed to evaluate the impact of “spin” (i.e., misrepresentation of study results) in health news stories reporting studies of pharmacologic treatments on patients’/caregivers’ interpretation of treatment benefit.

**Methods:**

We conducted three two-arm, parallel-group, Internet-based randomized trials (RCTs) comparing the interpretation of news stories reported with or without spin. Each RCT considered news stories reporting a different type of study: (1) pre-clinical study, (2) phase I/II non-RCT, and (3) phase III/IV RCT. For each type of study, we identified news stories reported with spin that had earned mention in the press. Two versions of the news stories were used: the version with spin and a version rewritten without spin. Participants were patients/caregivers involved in Inspire, a large online community of more than one million patients/caregivers. The primary outcome was participants’ interpretation assessed by one specific question “What do you think is the probability that ‘treatment X’ would be beneficial to patients?” (scale, 0 [very unlikely] to 10 [very likely]).

**Results:**

For each RCT, 300 participants were randomly assigned to assess a news story with spin (*n* = 150) or without spin (*n* = 150), and 900 participants assessed a news story. Participants were more likely to consider that the treatment would be beneficial to patients when the news story was reported with spin. The mean (SD) score for the primary outcome for abstracts reported with and without spin for pre-clinical studies was 7.5 (2.2) versus 5.8 (2.8) (mean difference [95% CI] 1.7 [1.0–2.3], *p* < 0.001); for phase I/II non-randomized trials, 7.6 (2.2) versus 5.8 (2.7) (mean difference 1.8 [1.0–2.5], *p* < 0.001); and for phase III/IV RCTs, 7.2 (2.3) versus 4.9 (2.8) (mean difference 2.3 [1.4–3.2], *p* < 0.001).

**Conclusions:**

Spin in health news stories reporting studies of pharmacologic treatments affects patients’/caregivers’ interpretation.

**Trial registration:**

ClinicalTrials.gov, NCT03094078, NCT03094104, NCT03095586

**Electronic supplementary material:**

The online version of this article (10.1186/s12916-019-1330-9) contains supplementary material, which is available to authorized users.

## Background

News stories represent an important source of information for patients [1–3]. In the USA, according to surveys of the Pew Research Center, 93% of the population reads at least some news online [4] and 67% declares following health news somewhat or very closely [5]. Furthermore, about two thirds of the time, reading health news prompts follow-up actions such as searching for more information or talking about it with others [6].

However, some evidence suggests that many news stories do not accurately represent research results and could mislead readers with “spin,” defined as “the presentation of information in a particular way, a slant, especially a favorable one” [7–12]. A systematic assessment of news stories highlighted in Google News showed that 88% of news stories were distorted (i.e., reported with spin) [13]. Several different types of spin could be used to distort the study results. The most frequent are misleading reporting such as not reporting adverse events, misleading interpretation such as claiming a causal effect despite the non-randomized study design, overgeneralization of the results such as extrapolating a beneficial effect from an animal study to humans, and highlighting a single-patient experience for the success of a new treatment instead of focusing on the group results [13].

Spin in news stories is often related to the presence of spin in the published article and its press release [9, 14]. A quantitative content analysis of 534 press releases and related research articles and news stories showed that the main source of spin was the press release [15]. A comparison of newspaper stories and peer-reviewed research papers in genetics showed that newspaper articles accurately conveyed the results, but there was an overemphasis on benefit and an underemphasis on risk in both the scientific article and related news story [16, 17].

The impact of spin has been rarely explored. A previous study showed that spin reported in abstracts of randomized controlled trials (RCTs) with a statistically non-significant primary outcome can affect trialists’ interpretations; the experimental treatments were rated more beneficial when the abstracts were reported with spin versus no spin [18]. To our knowledge, the impact of spin in health news stories on patients’/caregivers’ interpretation of the study results has not been evaluated in an experimental study.

The aim of this study was to evaluate the impact of spin in health news stories reporting various types of studies evaluating pharmacologic treatments on patients’/caregivers’ interpretation of the benefits of treatment.

## Methods

### Study design

We planned three Internet-based RCTs (ib-RCTs) comparing the interpretation of news stories reported with or without spin. We defined “spin” as a misrepresentation of study results, regardless of motive (intentionally or unintentionally), that overemphasizes the efficacy or overstates safety of the treatment as compared with that shown by the results [19]. Each RCT considered news stories reporting a different type of study evaluating pharmacologic treatments: (1) pre-clinical study, (2) phase I/II non-randomized trial, and (3) phase III/IV RCT. The protocol used for each RCT is detailed elsewhere [20].

### Participants

Participants were patients or caregivers involved in *Inspire*, a large online patient community (https://www.inspire.com). *Inspire* is a US-based company, founded in 2005, with a healthcare social network of more than one million patients and caregivers. Participants were eligible if they were members of the community and aged 18 years or older. They were invited by email to participate in an academic study investigating how medical research reporting affects how readers interpret and perceive health news stories (see invitation email in Additional file 1). Participants accessed the survey by using an Internet link included in the invitation email. The first page of the survey provided information on the study, and participants had to tick a box to consent to participate in the study (Additional file 2). They entered some demographic data and then were randomly assigned to read one news story with spin or one without spin. We sent invitation emails in waves until the planned number of participants logged on and completed the assessment. We sent a maximum of two reminders. Participants did not receive any monetary compensation.

### Identification of health news stories

We identified a sample of news stories with spin reporting studies of pharmacologic treatments that earned mention in the press or on social media (i.e., high Altmetric score). For this purpose, we searched *Altmetric Explorer* (https://www.altmetric.com) by using the *PubMed query* field in the advanced search. One researcher screened the retrieved citations sorted from the highest to lowest Altmetric score and assessed the related online news stories until the identification of 30 news stories reported with spin in the headline and text according to an existing classification [13]: 10 reporting pre-clinical studies, 10 reporting phase I/II non-randomized trials, and 10 reporting phase III/IV RCTs. For a given citation, when several news stories had spin, the researcher selected the news story with the most spin in the text. As a quality procedure, a second researcher confirmed the eligibility of all included news stories and screened 10% of the excluded news stories.

The search strategy and screening process are detailed in Additional file 3.

The reference list of the news stories and related publications are in Additional file 4.

### Interventions

We used two versions of news stories: the news story reported with spin and a rewritten version without spin.

We anonymized all news stories with and without spin by deleting information that could help identify the news story (date, name of the news outlet, journalist’s name, any reference to the original article, and trial). We used hypothetical names to conceal the names of pharmacological treatments, authors, and experts. We also used generic wording (profit or non-profit funding organizations) to mask the funders’ name when reported. We did not mask the research institution where the study was conducted if reported in the news story.

#### News story with spin

We kept the same structure and content of the original news story that was anonymized.

#### News story without spin

We rewrote all news stories reported with spin, this time without spin, keeping the same structure of the original news story. One researcher identified and deleted spin from the news stories according to pre-specified guidance (see Additional file 5). According to this guidance, when the news story did not report any limitation or caution, we added some caution according to the guidance reported in Table 1. As a quality control, one researcher checked the rewritten news stories. Disagreements were resolved by consensus or if necessary with a third researcher.

**Table 1:** Cautions added when appropriate by study type

Study type	Caution added when appropriate
Animal or laboratory study	“However, it may take years to know whether this treatment will be beneficial and safe for humans. In fact, less than 1% of the drugs tested on animals/cell culture are approved for clinical use in patients.”
Small study	“The treatment was tested on small number of patients; (…) Larger studies are needed to understand whether the treatment will be beneficial and safe.”
Uncontrolled study/lack of comparator	“Everyone in this study took this treatment. Without investigating patients who did not take this treatment, it is impossible to know whether taking this treatment accounted for the improved outcome or not. In fact, less than 10% of the drugs tested in preliminary clinical studies are approved for clinical use in patients. More research is needed to (…)”
Non-randomized study	“We do not know whether it was the treatment or something else that really accounted for the effect observed. In fact, less than 10% of the drugs tested in preliminary clinical studies are approved for clinical use in patients. More research is needed to (…)”
RCT	Cautions were reported according to the limitations of the published RCT or identified by the reviewer.

Depending on the study, some limitations could be added and the wording could be modified

*RCT* randomized controlled trial

### Outcomes

The primary outcome was participants’ interpretation of the benefits of treatment measured on a numerical rating scale (NRS) from 0 to10.

- What do you think is the probability that “treatment X” would be beneficial to patients? (scale, 0 [very unlikely] to 10 [very likely])

Secondary outcomes were as follows:

- What do you think is the size of the potential benefit of “treatment X” for patients? (4-point scale [none, small, moderate or large])
- How safe do you think that “treatment X” would be for patients? (NRS scale, 0 [very unsafe] to 10 [very safe])
- Do you think this treatment should be offered to patients in the short term? (NRS scale, 0 [absolutely no] to 10 [absolutely yes])
- Do you think this treatment will make a difference in existing clinical practice? (NRS scale, 0 [absolutely no] to 10 [absolutely yes])

### Sample size

Each participant read a news story with or without spin. For each RCT, we needed a sample of 266 assessments of news stories to detect an effect size of 0.4 with a power of 90% and *α* risk of 5% for each RCT [18]. Each news story was read the same number of times (balanced design), and we had to consider clustering because a news story would be read many times. To achieve this, we planned a sample size of 300 participants (150 in each group) for each RCT (i.e., an inflation factor of about 1.1). Therefore, each group assessed each news story 15 times (10 news stories with or without spin for 150 participants) for each RCT.

### Randomization and blinding

A statistician computer generated a random assignment sequence by using blocks of ten (i.e., number of news stories selected × 2) for each RCT. The use of a centralized online system ensured allocation concealment. Participants who logged on to the system were randomized. Participants who did not evaluate the news story allocated were excluded, and the news story was automatically allocated to another participant.

We informed all participants that they were participating in a survey about the interpretation of news stories reporting medical research that evaluates treatments but not about the objectives and hypothesis of the study. We told the participants that they would be informed of the results after completion of the study.

### Statistical analysis

Statistical analyses involved the use of R v2.15.1 (R foundation for Statistical Computing, Vienna, Austria). We analyzed the differences between groups by using a linear mixed model with fixed and random group effects and news stories × group interaction effects for each quantitative outcome in each RCT. Random effects allowed us to account for the following two levels of clustering: within-group clustering as a result of the news story (each news story assessed 15 times in each group) and between-group clustering (pairing between the news story used in the 2 arms of the trial). Inferences were based on the restricted maximum likelihood. For primary and secondary quantitative outcomes, we estimated the difference between means with 95% confidence intervals (CIs). We dichotomized one of the secondary outcomes assessed on a 4-point scale (i.e., size of the potential benefit for patients) as moderate or large versus none or small and analyzed differences between groups by a Poisson mixed model (using the same random effects described for the linear mixed model). We assessed the differences in qualitative outcome between groups by computing the relative risk with 95% CIs. *p* < 0.05 was considered statistically significant.

### Changes to the protocol

We initially planned to also perform an RCT of news stories reporting observational studies of pharmacologic treatments, but we failed to identify this type of news story. We modified the wording of the cautions added to the news story without spin from what was planned after pilot testing. We specified the definition of the secondary outcome: “What do you think is the size of the potential benefit of ‘treatment X’ for patients? (4-point scale [none, small, moderate or large])” as a dichotomous variable (moderate/large versus none/small) after the end of the trials but before the analysis.

## Results

### Participants

**Fig. 1:**
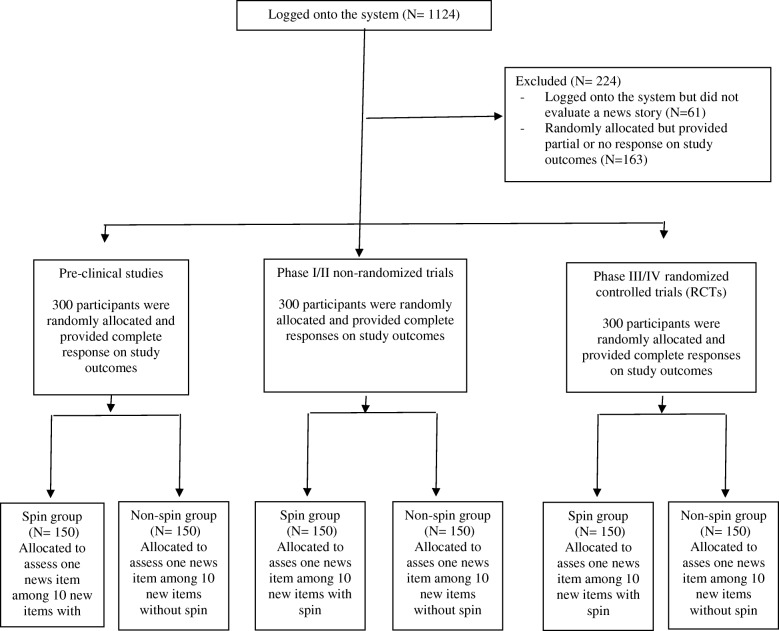
Flow of participants in the study

The study was launched on October 16, 2017, and completed on November 16, 2017. We sent an invitation email to 80,763 participants: 10027 opened the email, 1124 logged onto the system, and 224 were excluded because they did not evaluate the news story allocated; 900 evaluated the news stories allocated, and their data were analyzed (Fig. 1).

Overall, the participants were mainly women (> 80%) with age older than 50 years (Table 2). More than half declared they relied on news stories to make decisions about their health, and for about 40%, online health news was their preferred source of information on new treatments.

**Table 2:** Baseline characteristics of participants by study type

	Preclinical study			Phase I/II non-randomized trial			Phase III/IV RCT		
	Spin (*N* = 150)	No spin* (*N* = 150)	Total (*N* = 299)	Spin (*N* = 150)	No spin (*N* = 150)	Total (*N* = 300)	Spin (*N* = 150)	No spin (*N* = 150)	Total (*N* = 300)
Participants									
Patient	142 (94.6%)	138 (92.6%)	280 (93.7%)	134 (89.3%)	136 (90.7%)	270 (90%)	135 (90%)	137 (91.3%)	272 (90.7%)
Caregiver	4 (2.7%)	5 (3.4%)	9 (3.0%)	9 (6.0%)	8 (5.3%)	17 (5.7%)	10 (6.7%)	9 (6.0%)	19 (6.3%)
Others	4 (2.7%)	6 (4.0%)	10 (3.3%)	7 (4.7%)	6 (4.0%)	13 (4.3%)	5 (3.3%)	4 (2.7%)	9 (3.0%)
Age									
18–29	1 (0.7%)	5 (3.4%)	6 (2.0%)	5 (3.3%)	2 (1.3%)	7 (2.3%)	2 (1.3%)	3 (2.0%)	5 (1.6%)
30–49	26 (17.3%)	24 (16.1%)	50 (16.7%)	20 (13.3%)	22 (14.7%)	42 (14.0%)	29 (19.4%)	27 (18.0%)	56 (18.7%)
50–69	88 (58.7%)	90 (60.4%)	178 (59.5%)	95 (63.4%)	90 (60.0%)	185 (61.7%)	102 (68.0%)	90 (60.0%)	192 (64.0%)
≥ 70	35 (23.3%)	30 (20.1%)	65 (21.7%)	30 (20.0%)	36 (24.0%)	66 (22.0%)	17 (11.3%)	30 (20.0%)	47 (15.7%)
Sex (female)	123 (82.0%)	119 (79.9%)	242 (80.9%)	143 (95.3%)	134 (89.3%)	277 (92.3%)	120 (80.0%)	125 (83.3%)	245 (81.7%)
Frequency of reading news stories									
Never	1 (0.7%)	3 (2.0%)	4 (1.3%)	0 (0.0%)	1 (0.7%)	1 (0.3%)	1 (0.7%)	1 (0.7%)	2 (0.7%)
1/month	4 (2.7%)	4 (2.7%)	8 (2.7%)	9 (6.0%)	9 (6.0%)	18 (6.0%)	4 (2.7%)	2 (1.3%)	6 (2.0%)
1/week	14 (9.3%)	17 (11.4%)	31 (10.4%)	21 (14.0%)	10 (6.7%)	31 (10.3%)	15 (10.0%)	20 (13.3%)	35 (11.7%)
Daily	131 (87.3%)	125 (83.9%)	256 (85.6%)	120 (80.0%)	130 (86.6%)	250 (83.4%)	130 (86.6%)	127 (84.7%)	257 (85.6%)
Rely on health news stories to make decisions about health	86 (57.3%)	80 (53.7%)	166 (55.5%)	88 (58.7%)	98 (65.3%)	186 (62.0%)	89 (59.3%)	83 (55.3%)	172 (57.3%)
Primary source of information about new treatments									
Online health news	62 (41.3%)	62 (41.6%)	124 (41.5%)	76 (50.7%)	68 (45.3%)	144 (48.0%)	65 (43.3%)	55 (36.7%)	120 (40.0%)
Physicians	63 (42.0%)	59 (39.6%)	122 (40.8%)	44 (29.3%)	50 (33.3%)	94 (31.3%)	55 (36.7%)	62 (41.3%)	117 (39.0%)
Family or friends	1 (0.7%)	1 (0.7%)	2 (0.7%)	4 (2.7%)	2 (1.4%)	6 (2.0%)	3 (2.0%)	1 (0.7%)	4 (1.3%)
Television	1 (0.7%)	2 (1.3%)	3 (1.0%)	1 (0.7%)	0 (0.0%)	1 (0.3%)	1 (0.7%)	4 (2.7%)	5 (1.7%)
Social media	7 (4.7%)	8 (5.4%)	15 (5.0%)	8 (5.3%)	6 (4.0%)	14 (4.7%)	12 (8.0%)	6 (4.0%)	18 (6.0%)
Others	16 (10.6%)	17 (11.4%)	33 (11.0%)	17 (11.3%)	24 (16.0%)	41 (13.7%)	14 (9.3%)	22 (14.6%)	36 (12.0%)

*RCT* randomized controlled trial

*The baseline data were missing for one participant

### Interventions

Additional file 6 reports the spin identified in the news stories and the modifications. Overall, all headlines were reported with spin and were modified. All news stories exhibited misleading reporting. Particularly, we identified and deleted linguistic spin in 29 news stories and added some cautions in all news stories. Misleading interpretation was mainly identified for phase I/II non-randomized trials (*n* = 10) and phase III/IV RCTs (*n* = 7), and inadequate extrapolation was reported in 26 news stories. Finally, we identified and modified or deleted interviews with the investigator/expert in 18 news stories and the patient in 1 story. News stories reported with and without spin are reported in Additional file 7.

### Outcomes

**Fig. 2:**
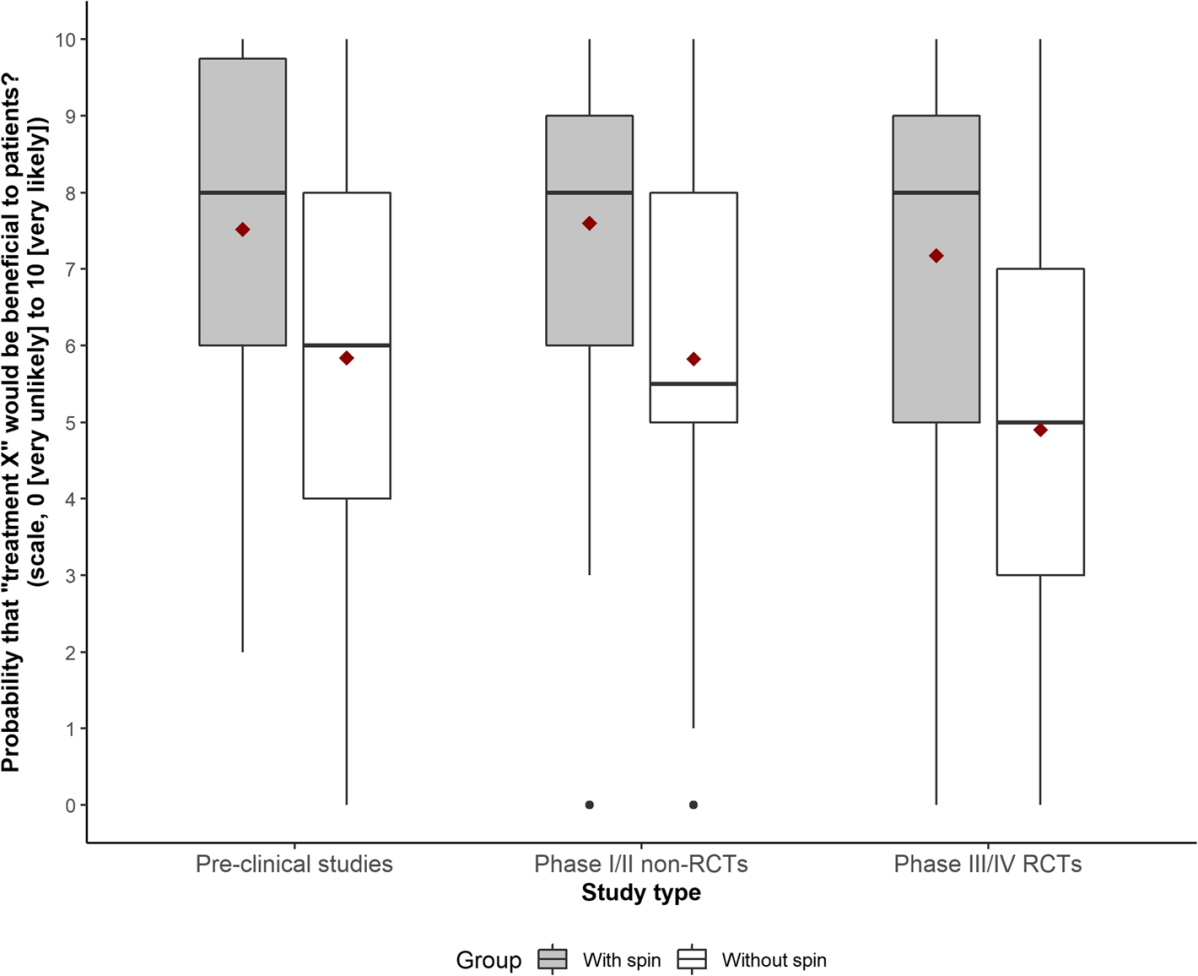
Participants’ interpretation of the benefit of treatments when reading a news story reported with or without spin. Scores are based on a numerical rating scale, ranging from 0 (very unlikely) to 10 (very likely). Boxes represent median observations (horizontal rule) with 25th and 75th percentiles of observed data (box edges). The diamonds represent the mean. The error bars represent the minimum and maximum values. RCTs, randomized controlled trials

**Fig. 3:**
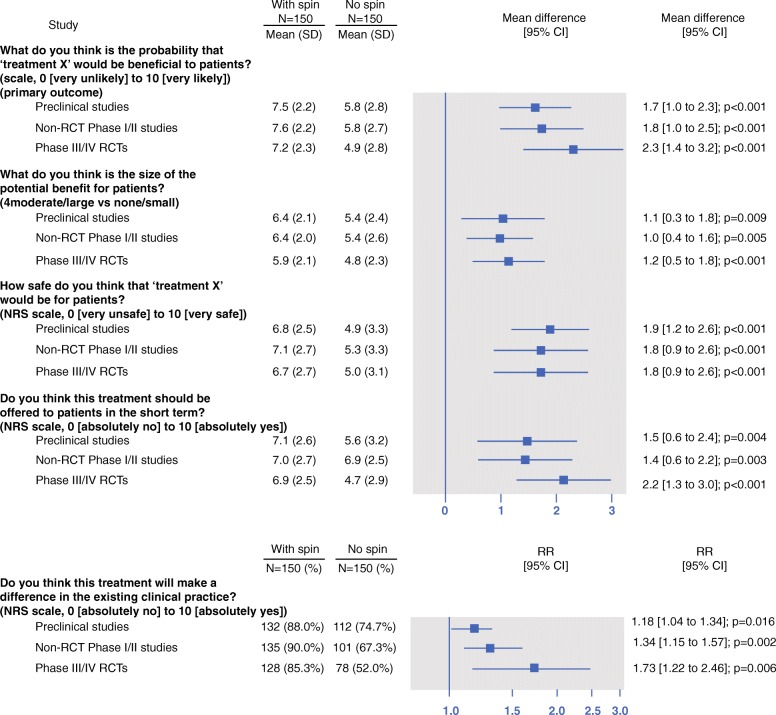
Forest plot of the results for primary and secondary outcomes

Overall, whatever the study design reported in the news stories, participants reading a news story with spin were more likely to believe that the treatment would be beneficial for them. The mean (SD) score for the primary outcome for abstracts reported with and without spin for pre-clinical studies was 7.5 (2.2) versus 5.8 (2.8) (mean difference [95% CI] 1.7 [1.0–2.3], *p* < 0.001); for phase I/II non-randomized trials, 7.6 (2.2) versus 5.8 (2.7) (mean difference 1.8 [1.0–2.5], *p* < 0.001); and for phase III/IV RCTs, 7.2 (2.3) versus 4.9 (2.8) (mean difference 2.3 [1.4–3.2], *p* < 0.001) (Fig. 2; Fig. 3; Additional file 8). Results were consistent for secondary outcomes (Fig. 3; Additional file 8).

## Discussion

The results of these 3 ib-RCTs involving 900 participants showed that spin in news stories can affect the interpretation of the benefit of a treatment: participants were more likely to believe the treatment was beneficial when news stories were reported with spin.

To our knowledge, this is the first time that the RCT design has been used to explore the impact of spin in news stories. A previous RCT explored the impact of spin in abstracts of published reports of RCTs evaluating treatment in the field of cancer on interpretation by trialists in this field [18]. This trial found that spin could affect trialists’ interpretations, but the effect estimate was lower. A possible explanation could be the higher experience of trialists in interpreting research, the lower frequency of spin in abstracts than news stories, and the better reporting in abstracts, providing data on the design, sample size, and treatment effect estimates, which could affect readers’ interpretation.

Previous studies of spin in news stories showed the high prevalence of spin in research articles [19, 21–23], in press releases, and in news stories [13]. The HealthNewsReview.org project launched in 2006 critically appraises news stories and press releases. For a large sample of health news stories, it showed that most stories were graded unsatisfactory; two thirds failed to adequately address cost and quantify harms and benefit [24]. Frequent misleading reporting included stories emphasizing benefits, a focus on relative instead of absolute risk reduction; relying on anecdotes; and omitting harms and study limitations [24, 25]. However, the presence of spin in news stories is not solely the responsibility of journalists. Evidence showed that spin in news stories was related to the misrepresentation of study results in the research report and press release [9, 14, 16, 17, 26].

It is important to interpret our results considering the complexity of the ecosystem leading to research communication [27, 28]. This system, involving several stakeholders (researchers, peer-reviewers, editors, funders, institutions, and the public) highly driven by competition, is producing a “cycle of spin” [29]. Indeed, several forces acting on various stakeholders are contributing to spin [16, 29]; these forces are related to pressure to publish, to obtain citations, to obtain media coverage, and to attract the public’s attention [29].

Misinterpreting the content of news stories because of spin could have important public health consequences because the mass media can affect patient and public behavior. An interrupted time series analysis with UK primary care data showed an increased likelihood of stopping treatment associated with the publication of controversial articles about statins that were widely disseminated in the media, which, according to the authors, could result in 2000 extra cardiovascular events [30]. However, this interpretation has been questioned [31]. An animal and early phase study evaluating lithium for amyotrophic lateral sclerosis [32] led to its rapid use by patients despite a lack of evidence in humans. The lack of effect of this treatment was secondarily demonstrated [33]. A Cochrane systematic review concluded that mass media interventions may have an important role in influencing the use of health care interventions [2]. In April 2018, HealthNewsReview.org launched a new series dedicated to “Patient harm from misleading media” that provides examples of harm occurring when people believe and act on what they read in news with spin.

This study has several strengths. We explored the different types of news stories about studies receiving a lot of public attention. The studies and treatments evaluated were diverse. The news stories were anonymized, and participants were blinded to the study hypothesis to avoid evaluation bias. The process to delete spin and add some cautions was standardized and followed specific guidance. Participants were members of a large US online patient support community who frequently read news stories and relied on news stories to make decisions about their health. The results were consistent whatever the study design reported in the news story.

Our study also has some limitations, and the results should be interpreted with caution. Particularly, we evaluated only written news stories and cannot extrapolate our results to other mass media. Furthermore, participants were not specifically concerned by the content of the news story, and we could not explore whether they would change their behavior after reading the news story. Finally, the response rate was low and consisted of mainly females which reflects the high participation of females in Inspire and in most health online communities. This prevalence could affect the external validity of our results. However, more than half of our participants declared that they relied on news stories to make decisions about their health, and about 40% used online health news as their preferred source of information on new treatments. Further, a survey performed by the American Press Institute and the Associated Press-NORC Center for Public Affairs Research showed that women more closely follow stories about health and medicine than do men [34]. Consequently, our study targeted the population most likely to be exposed to news stories.

## Conclusion

Our results show that spin in health news stories can affect the interpretation of study results. Research communication relies on a complex interactive ecosystem involving several stakeholders and various forces that feeds a “cycle of spin” [29]. In an era where trustful and effective science communication is essential, we need to rethink and change the current ecosystem. Researchers and institutions should move from the “publish or perish” model to a model in which researchers make every effort to avoid distortion and hype [35]. Researchers should be specifically trained to understand how citizens use the media and consequently frame their research communication to the public in a way which is truthful and relevant for the different audiences [36]. Journalists must realize the harm that can be caused when they fail to detect spin and promote it to their readers. Training is available to help them improve their reporting on research [37]. News consumers can also access tools to improve their own critical analysis of claims. Finally, research in this field should be reinforced; a research agenda on this topic was proposed by the US National Academy of Science [38]. This agenda particularly highlights the need for a system approach to research communication.

## Additional files

Additional file 1: Invitation email. (DOCX 14 kb)

Additional file 2: Information and consent. (DOCX 256 kb)

Additional file 3: Identification of news stories reported with spin. Search strategy. (DOCX 14 kb)

Additional file 4: References list of the 30 news stories and articles. (DOCX 57 kb)

Additional file 5: Guidance to rewrite news story without spin. (DOCX 19 kb)

Additional file 6: Description of spin and modifications performed in the rewritten news stories without spin by study type. (DOCX 21 kb)

Additional file 7: News story reported with and without spin. (DOCX 69 kb)

Additional file 8: Outcomes by study type. (DOCX 17 kb)

## Abbreviations

CI: Confidence interval
ib-RCT: Internet-based randomized controlled trial
NRS: Numerical rating scale
RCT: Randomized controlled trial
