# Correction to: Three randomized controlled trials evaluating the impact of “spin” in health news stories reporting studies of pharmacologic treatments on patients’/caregivers’ interpretation of treatment benefit

**DOI:** 10.1186/s12916-019-1388-4

**Published:** 2019-07-27

**Authors:** Isabelle Boutron, Romana Haneef, Amélie Yavchitz, Gabriel Baron, John Novack, Ivan Oransky, Gary Schwitzer, Philippe Ravaud

**Affiliations:** 1 INSERM, UMR 1153, Epidemiology and Biostatistics Research Center (CRESS), Methods Team, Paris, France; 2 Faculté de Médecine, Paris Descartes University, Paris, France; 3 Centre d’Épidémiologie Clinique, AP-HP (Assistance Publique des Hôpitaux de Paris), Hôpital Hôtel Dieu, 1, Place du parvis Notre Dame, 75004 Paris Cedex 4, France; 4 Inspire, Arlington, VA USA; 5 New York University’s Arthur Carter Journalism Institute, New York, USA; 6 HealthNewsReview.org, University of Minnesota, School of Public Health, Minneapolis, MN USA; 7 Department of Epidemiology, Columbia University Mailman School of Public Health, New York, NY USA

**Correction to: BMC Med (2019) 17:105**

**https://doi.org/10.1186/s12916-019-1330-9**

Figure 3 in the original article [1] is incorrect; labels for secondary outcomes have been shifted and do not correspond to the numbers reported in the table (Additional File 8).

The corrected version can be seen ahead.

This figure should be used over the Fig. 3 seen in the original article.

This error does not affect the results, interpretation, or conclusion.

**Fig. 3:**
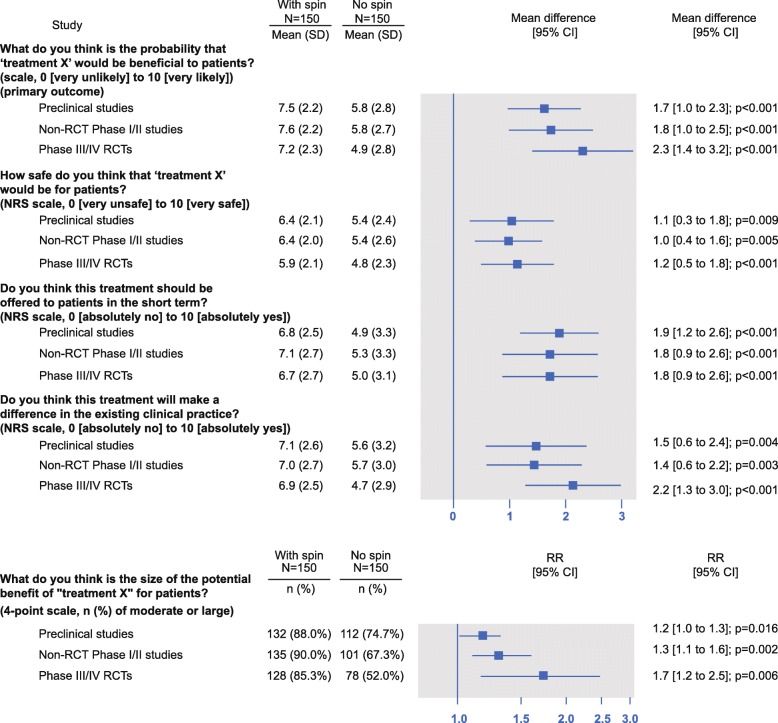
Forest Plot of the results for primary and secondary outcomes
