# Supplementary material for: Three randomized controlled trials evaluating the impact of “spin” in health news stories reporting studies of pharmacologic treatments on patients’/caregivers’ interpretation of treatment benefit
Source: BMC Med. 2019 Jun 4;17:105. doi: 10.1186/s12916-019-1330-9 (PMC6547451; doi:10.1186/s12916-019-1330-9)
Supplement: Supplementary file 1 — Invitation email. (DOCX 14 kb) [file 12916_2019_1330_MOESM1_ESM.docx]

**Additional file 1:** Invitation email.

Subject: How is medical research reported in health news interpreted by general public? An academic study

On behalf of the team at Inspire, we invite you to participate in an international academic study to investigate how medical research reporting impacts the way we interpret and perceive health news items.

Together with our partners, we have created an online survey and should take about 5 minutes to complete. The survey involves reading an excerpt from a news article and answering five short questions.

Your participation in this study will help us to improve the communication of medical research in health news for patients, caregivers, and the public.

Be assured that Inspire understands that your personal and health information is private. All answers will be treated confidentially, and no personal information about you will be shared with anyone. This study has been approved by INSERM, Institutional Review Board (IRB 00003888).

We will share the results of this study with you upon its completion.

Click here to take the survey: XX

Or copy and paste the following link into your web browser: XX

Thank you for your participation.

With best wishes

Kathryn Ticknor, Sr. Research Manager, Inspire

Dave Taylor, Sr. Director of research, Inspire

Isabelle Boutron, Prof. Epidemiology, University Paris Descartes, INSERM

Ivan Oransky, New York University's Arthur Carter Journalism Institute

Gary Schwitzer, Associate Professor, University of Minnesota School of Public Health

Inspire Logo to the survey homepage
