# Supplementary figures and images for: Three randomized controlled trials evaluating the impact of “spin” in health news stories reporting studies of pharmacologic treatments on patients’/caregivers’ interpretation of treatment benefit
Source: BMC Med. 2019 Jun 4;17:105. doi: 10.1186/s12916-019-1330-9 (PMC6547451; doi:10.1186/s12916-019-1330-9)

**Additional file 2:** Information and consent.


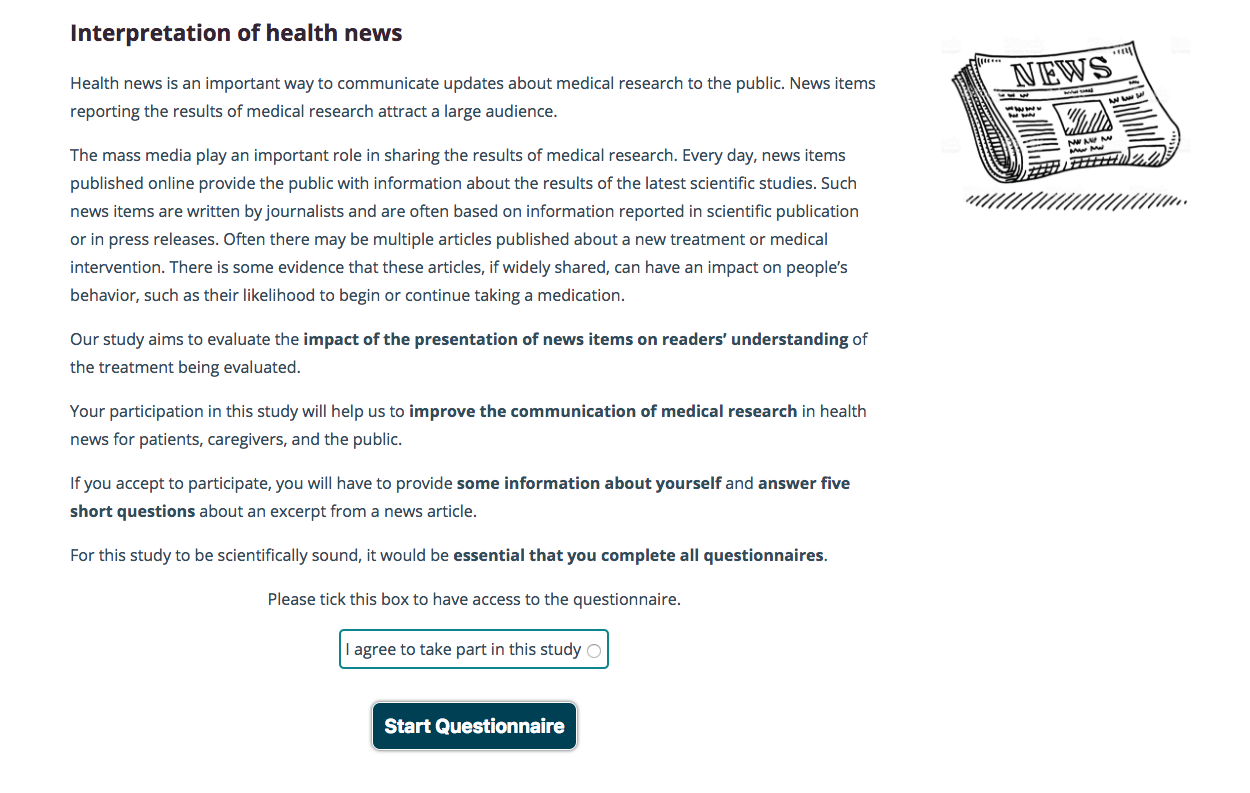

Supplement: Supplementary file 2 — Information and consent. (DOCX 256 kb) [file 12916_2019_1330_MOESM2_ESM.docx]
