# Supplementary material for: Three randomized controlled trials evaluating the impact of “spin” in health news stories reporting studies of pharmacologic treatments on patients’/caregivers’ interpretation of treatment benefit
Source: BMC Med. 2019 Jun 4;17:105. doi: 10.1186/s12916-019-1330-9 (PMC6547451; doi:10.1186/s12916-019-1330-9)
Supplement: Supplementary file 3 — Identification of news stories reported with spin. Search strategy. (DOCX 14 kb) [file 12916_2019_1330_MOESM3_ESM.docx]

**Additional file 3:** Identification of news stories reported with spin. Search strategy.

Search strategy on Altmetric explorer using the PubMed query field in the advanced search page ((Randomized controlled trial[Publication Type] OR Observational study[Publication Type]) OR Meta-analysis[Publication Type]) OR Randomized[Title/Abstract]) OR controlled[Title/Abstract]) OR trial[Title/Abstract]) OR cross-sectional[Title/Abstract]) OR case-control[Title/Abstract]) OR cohort[Title/Abstract]) OR Meta-analysis[Title/Abstract]) OR systematic review[Title/Abstract]) AND (hasabstract[text] AND ("2014/01/01"[PDAT] : "2014/06/30"[PDAT]))) OR Cell culture study[Title/Abstract]) OR Animal study[Title/Abstract]) OR pre-clinical study[Title/Abstract] AND (hasabstract[text] AND ("animals"[MeSH Terms:noexp] OR "humans"[MeSH Terms]))

Screening process

Screening was performed in two steps:

First, one researcher screened the citations sorted from highest to lowest Altmetric score to identify studies evaluating the effect of a pharmacological treatment, regardless of study design and study population (including human and animal/laboratory). For each study fulfilling eligibility criteria, the researcher retrieved the published article and all related online news stories available at *Altmetric Explorer*.

Second, the researcher identified news items with spin in the headline and text by using a standard scheme of spin as detailed in the table below. When several news stories had spin in the headline, the researcher selected the news item with the most spin in the text. We included only news stories reported by general or medical news outlets or lay press whose target consumers were the general population.
