# Supplementary material for: Three randomized controlled trials evaluating the impact of “spin” in health news stories reporting studies of pharmacologic treatments on patients’/caregivers’ interpretation of treatment benefit
Source: BMC Med. 2019 Jun 4;17:105. doi: 10.1186/s12916-019-1330-9 (PMC6547451; doi:10.1186/s12916-019-1330-9)
Supplement: Supplementary file 4 — References list of the 30 news stories and articles. (DOCX 57 kb) [file 12916_2019_1330_MOESM4_ESM.docx]

**Additional file 4:** Reference list of the 30 news stories and articles

|  |  |  |  |  |  |  |  |  |  | **Word count** | |
| --- | --- | --- | --- | --- | --- | --- | --- | --- | --- | --- | --- |
| **S/NO** | **Altmetric score** | **Journal Name** | **PMID** | **Title** | **Medical area/condition** | **Pharmacological treatment** | **Hypothetical names of the drugs** | **Headlines of the selected news items** | **Electronic link to the news items** | **News items with spin** | **News items without spin** |
| **Pre-clinical studies** | |  |  |  |  |  |  |  |  |  |  |
| 1 | 321 | PNSS | 24395803 | TRAIL-coated leukocytes that kill cancer cells in the circulation. | Cancer | Sticky balls (i.e., Cancer-killing protein called TRAIL) | - | New "sticky balls" that can prevent cancer spread | http://www.business-standard.com/article/news-ani/now-sticky-balls-that-can-prevent-cancer-spread-114010800642_1.html | 178 | 221 |
| 2 | 248 | Science Translational Medicine | 24431111 | Therapeutic inflammatory monocyte modulation using immune-modifying microparticles. | Inflammatory conditions | Microparticles | - | Microparticles may reduce heart attack damage | http://www.thehindu.com/sci-tech/health/medicine-and-research/microparticles-may-reduce-heart-attack-damage/article5582414.ece | 246 | 167 |
| 3 | 181 | Biometrials | 24161167 | The enhancement of bone regeneration by gene activated matrix encoding for platelet derived growthfactor. | Bone regeneration | Bio-patch | - | Regrowing Bones: "Bio Patch" Restores Bones Tissues at Damaged, Weakened Sites | http://www.medicaldaily.com/regrowing-bones-bio-patch-restores-bone-tissue-damaged-weakened-sites-262344 | 411 | 370 |
| 4 | 130 | Biometrials | 24094935 | InÂ vivo performance of a drug-eluting contact lens to treat glaucoma for a month | Glaucoma | Latanoprost | Frosan | Contact lenses can deliver glaucoma drugs for a month | http://www.medicalnewstoday.com/articles/269899.php | 526 | 463 |
| 5 | 76 | PNAS | 24982170 | Engineered nanomedicine for myeloma and bone microenvironment targeting | Cancer/Myeloma | Nanoparticles with Alendronate | Nanoparticles with Tebroid | New Nanoparticles Simultaneously Kill Cancer And Strengthen Bones | http://www.meddeviceonline.com/doc/new-nanoparticles-simultaneously-kill-cancer-and-strengthen-bones-0001 | 427 | 367 |
| 6 | 75 | ACS Nano | 24506583 | Diamond Nanogel-Embedded Contact Lenses Mediate Lysozyme-Dependent Therapeutic Release | Glaucoma | Nanodiamond with timol maleate | Nanodiamond with elatin | Novel contact lenses "enable more effective glaucoma drug delivery" | http://www.medicalnewstoday.com/articles/272708.php | 535 | 537 |
| 7 | 68 | Biometrials | 24210875 | Nanoparticle-directed sub-cellular localization of doxorubicin and the sensitization breast cancer cells by circumventing GST-Mediated drug resistance | Breast cancer | Nanoparticles with doxirubicin | Nanoparticles with zorid | Nanoparticles with chemotherapeutic drugs can kill drug-resistant breast cancer cells | http://www.news-medical.net/news/20131108/Nanoparticles-with-chemotherapeutic-drugs-can-kill-drug-resistant-breast-cancer-cells.aspx | 318 | 310 |
| 8 | 64 | Nanomedicine: Nanotechnology, Biology and Medicine | 24566274 | Local hyperthermia treatment of tumors induces CD8+ T cell-mediated resistance against distal and secondary tumors | Tumors/cancer | Nanoparticles | - | Nanoparticles at specific temperature stimulate antitumor response | http://www.medicalnewstoday.com/releases/292445.php | 407 | 438 |
| 9 | 56 | Diabetes | 24379349 | GLP-1/glucagon co-agonism restores leptin responsiveness in obese mice chronically maintained on an obesogenic diet | Obesity | Glucagon+Leptin; GLP-1/Glucagon | Dicafen+Leptin; DLP-1/Dicafen | Metabolic damage from high calorie diet reversed by hormone | http://foodexposed.co.za/metabolic-damage-from-high-calorie-diet-reversed-by-hormone/ | 265 | 282 |
| 10 | 43 | Foot & Ankle International | 24403347 | Stem Cell-Bearing Suture Improves Achilles Tendon Healing in a Rat Model | Achilles tendinitis | Stem cells | Stem cells | Stem cells inside sutures could improve healing in Achilles tendon injuries | https://medicalxpress.com/news/2014-03-stem-cells-sutures-achilles-tendon.html?src_id=alt | 273 | 256 |
| **Phase I/II trials** | |  |  |  |  |  |  |  |  |  |  |
| 1 | 174 | The Lancet | 24412048 | Long-term safety and tolerability of ProSavin, a lentiviral vector-based gene therapy for Parkinson's disease: a dose escalation, open-label, phase 1/2 trial | Parkinson | Dopamine & ProSavin | Adofine & Deo Va | Novel gene therapy for Parkinson’s disease clears safety hurdle | http://www.rawstory.com/2014/01/novel-gene-therapy-for-parkinsons-disease-clears-safety-hurdle/ | 371 | 489 |
| 2 | 137 | The Lancet | 24503266 | Autologous mesenchymal stromal cell infusion as adjunct treatment in patients with multidrug and extensively drug-resistant tuberculosis: an open-label phase 1 safety trial. | Tuberculosis | Autologuos mesenchymal stromal cell infusion | Stem cells | Multi-Drug Resistant Tuberculosis Treatment 3 Times More Effective With Stem Cells | http://www.medicaldaily.com/multi-drug-resistant-tuberculosis-treatment-3-times-more-effective-stem-cells-266658 | 729 | 585 |
| 3 | 105 | NEJM | 24450858 | PI3KÎ´ Inhibition by Idelalisib in Patients with Relapsed Indolent Lymphoma. | Lymphoma | Idelalisib | Delibel | New Drug Shows Promise Against Lymphoma | http://www.newsmax.com/health/Health-News/lymphoma-drug-idelalisib/2014/01/23/id/548687/ | 333 | 375 |
| 4 | 89 | Clinical Infectious Disease | 24723284 | Immunogenicity and Safety of the Quadrivalent Human Papillomavirus Vaccine in HIV-1-Infected Women | Human papillomavirus infection | Human Pappilomavirus Vaccine (Gardasil) | Human Pappilomavirus Vaccine | Cancer Vaccine Proves Effective in H.I.V. Patients | http://www.nytimes.com/2014/04/22/health/cancer-vaccine-proves-effective-in-hiv-patients.html?_r=0 | 311 | 314 |
| 5 | 84 | Lancet Oncology | 24332512 | Safety and Activity of PD1 Blockade by Pidilizumab in Combination with Rituximab in Patients with Relapsed Follicular Lymphoma: a Single Group, Open-label, Phase 2 Trial | Lymphoma | Pidilizumab & Rituximab | Uxirti & Zubidel | TWO - DRUG COMBO COULD HELP TREAT CANCER | http://www.dnaindia.com/health/report-two-drug-combo-could-help-treat-cancer-1933910 | 270 | 331 |
| 6 | 58 | Journal of Clinical Oncology | 24493717 | Open-Label, Exploratory Phase II Trial of Oral Itraconazole for the Treatment of Basal Cell Carcinoma | Basal Cell Carcinoma | Oral Itraconazole | Bitrafin | Oral Antifungal Drug Could Help Treat Common Skin Cancer, Stanford Researchers Say | http://www.natureworldnews.com/articles/5872/20140204/oral-antifungal-drug-help-treat-common-skin-cancer-stanford-researchers.htm | 299 | 432 |
| 7 | 70 | Allergy, Asthma & Clinical Immunology | 24576338 | Phase 1 results of safety and tolerability in a rush oral immunotherapy protocol to multiple foods using Omalizumab. | | Omalizumab | Zumanil | Asthma Drug Helps Desensitize People To Multiple Food Allergens At Once | http://www.redorbit.com/news/health/1113084920/food-allergy-treatment-with-asthma-drug-030114/ | 528 | 649 |
| 8 | 57 | Journal of Clinical Oncology | 24711551 | Phase I Trial of a Novel Anti-GD2 Monoclonal Antibody, Hu14.18K322A, Designed to Decrease Toxicity in Children With Refractory or Recurrent Neuroblastoma | Neuroblastoma | Monoclonal antibody (Hu14.18K322A) | Monoclonal antibody Stubizil | Experimental antibody shows early promise for treatment of childhood tumor | http://www.healthcanal.com/cancers/50627-experimental-antibody-shows-early-promise-for-treatment-of-childhood-tumor.html?fulltext=true | 725 | 746 |
| 9 | 57 | Human Gene Therapy | 24524415 | Intracerebral Administration of Adeno-Associated Viral Vector Serotype rh.10 Carrying Human SGSH and SUMF1 cDNAs in Children with Mucopolysaccharidosis Type IIIA Disease: Results of a Phase I/II Trial | Mucopolysaccharidosis Type III A | Viral vector serotype/ Gene therapy | - | Gene Therapy for Lysosomal Storage Disease Shown to Be Safe and Well Tolerated, with Promising Results | http://www.healthcanal.com/genetics-birth-defects/48535-gene-therapy-for-lysosomal-storage-disease-shown-to-be-safe-and-well-tolerated-with-promising-results.html | 213 | 249 |
| 10 | 56 | Journal of Alternative & Complementary Medicine | 24635447 | Dextrose and Morrhuate Sodium Injections (Prolotherapy) for Knee Osteoarthritis: A Prospective Open-Label Trial | Osteoarthritis | Prolotherapy | - | New nonsurgical approach to treat chronic pain and stiffness linked with knee osteoarthritis | http://www.news-medical.net/news/20140409/New-nonsurgical-approach-to-treat-chronic-pain-and-stiffness-linked-with-knee-osteoarthritis.aspx | 142 | 287 |
| **RCTs** |  |  |  |  |  |  |  |  |  |  |  |
| 1 | 674 | The Lancet | 24485709 | Assessing the efficacy of oral immunotherapy for the desensitisation of peanut allergy in children (STOP II): a phase 2 randomised controlled trial | Peanut allergy | Oral Immunotherapy (OIT) | - | Success for trial of peanut allergy therapy | http://www.scienceworldreport.com/articles/12535/20140130/breakthrough-treatment-for-children-with-peanut-allergies.htm | 486 | 489 |
| 2 | 372 | The Lancet | 24655729 | Effect of high-dose simvastatin on brain atrophy and disability in secondary progressive multiple sclerosis (MS-STAT): a randomised, placebo-controlled, phase 2 trial | Multiple sclerosis | Simvastatin (Zocor) | Matisavit (Lisot) | Cholesterol Lowering Drug Slows Progression of Advanced Multiple Sclerosis | http://www.ibtimes.co.in/cholesterol-lowering-drug-slows-progression-of-advanced-multiple-sclerosis-544002 | 415 | 410 |
| 3 | 326 | JAMA | 24549548 | Effect of Citalopram on Agitation in Alzheimer's Disease: The CitAD Randomized Controlled Trial | Alzheimer | Citalopram (celex & cipramil) | Frabic (Febex & Feraxil) | Citalopram Can Reduce Alzheimer’s Agitation | http://psychcentral.com/news/2014/02/20/citalopram-can-reduce-alzheimers-agitation/66160.html | 386 | 379 |
| 4 | 222 | NEJM | 24897083 | Single-dose oritavancin in the treatment of acute bacterial skin infections. | Bacterial skin infections | Oritavancin & Vancomycin | Zubinil & Orabiol | Single Shot of Oritavancin Effective in Treating MRSA | http://www.natureworldnews.com/articles/7414/20140605/single-shot-oritavancin-effective-treating-mrsa.htm | 351 | 372 |
| 5 | 220 | The Lancet | 24183563 | Pimavanserin for patients with Parkinson's disease psychosis: a randomised, placebo-controlled phase 3 trial | Parkinson | Pimavanserin | Erifril | A drug that could change the way we treat Parkinson's disease just got approved | https://uk.finance.yahoo.com/news/drug-could-change-way-treat-123815638.html | 276 | 335 |
| 6 | 197 | NEJM | 24881463 | Adjuvant Exemestane with Ovarian Suppression in Premenopausal Breast Cancer. | Breast cancer | Exemestane & Tamoxifen | Elensin & Tiosin | Drug offers hope in fight against breast cancer | http://www.heraldsun.com.au/news/victoria/hormone-therapy-reduces-chances-of-recurring-breast-cancer-says-new-research/story-fni0fit3-1226940124384 | 271 | 307 |
| 7 | 196 | NEJM | 24450857 | Idelalisib and Rituximab in Relapsed Chronic Lymphocytic Leukemia. | Lymphotic leukemia | Idelalisib & Rituximab | Dalibel & Uxirit | New pill can 'melt away' leukemia without chemotherapy | http://www.business-standard.com/article/news-ani/new-pill-can-melt-away-leukemia-without-chemotherapy-114012400227_1.html | 308 | 324 |
| 8 | 194 | JAMA | 24756515 | Lorazepam vs Diazepam for Pediatric Status Epilepticus: A Randomized Clinical Trial | Epilepticus | Lorazepam (Ativan) & Diazepam (Valium) | Cirale (Kisfar) & Zorale (Udal) | Two Commonly Used Medications Equally Effective in Treating Seizures in Children | http://www.healthcanal.com/child-health/50090-two-commonly-used-medications-equally-effective-in-treating-seizures-in-children.html | 559 | 646 |
| 9 | 167 | NEJM | 24521108 | Comparison of pregabalin with pramipexole for restless legs syndrome. | Restless legs syndrome | Pregabalin (Lyrica) & Prmipexole (Mirepex) | Linapeg (Bynca) & Blinexole (Trizofil) | New Drug Shows Promise for Restless Legs Syndrome | http://www.technology.org/2014/02/14/new-drug-shows-promise-restless-legs-syndrome/ | 254 | 409 |
| 10 | 112 | JAMA | 24756514 | Effect of acetazolamide on visual function in patients with idiopathic intracranial hypertension and mild visual loss: the idiopathic intracranial hypertension treatment trial. | Idiopathic intracranial hypertension | Acetazolamide (Diamox) | Udafen (Smitra) | Glaucoma drug helps women with blinding disorder linked to obesity | http://now.uiowa.edu/2014/04/glaucoma-drug-helps-women-blinding-disorder-linked-obesity | 236 | 302 |
