# Supplementary material for: Three randomized controlled trials evaluating the impact of “spin” in health news stories reporting studies of pharmacologic treatments on patients’/caregivers’ interpretation of treatment benefit
Source: BMC Med. 2019 Jun 4;17:105. doi: 10.1186/s12916-019-1330-9 (PMC6547451; doi:10.1186/s12916-019-1330-9)
Supplement: Supplementary file 5 — Guidance to rewrite news story without spin. (DOCX 19 kb) [file 12916_2019_1330_MOESM5_ESM.docx]

**Additional file 5**: Guidance to rewrite news story without spin

| Type of spin* | Guidance |
| --- | --- |
| *Misleading information in headline* | Delete the misleading information and report the appropriate information. |
| *Misleading reporting (i.e., incomplete or inadequate reporting of any important information in the context of the research that could be misleading for the reader)* |  |
| - Misleading reporting of study design | Report the appropriate study design. |
| - Not reporting study population if an animal study | Report animal study subjects |
| - Selective reporting of outcomes favoring the beneficial effect of the treatment (e.g., statistically significant results for efficacy outcomes or statistically non-significant results for safety outcomes) | Report the results for all primary outcomes. |
| - Not reporting adverse events | Report adverse events when higher in one group.  (particularly frequent and serious adverse events related to treatment primarily.) |
| - Use of linguistic spin (i.e., any word or expression emphasizing the beneficial effect of the treatment) | Delete linguistic spin. |
| - Not reporting study limitations - Not reporting any caution about study design and results | Report study limitations and cautions with standardized text as reported in box 2. |
| - Any other type of misleading reporting not classified under the above section | Delete spin |
| *Misleading interpretation (i.e., interpretation of the study results in news stories that is not consistent with the results reported in the scientific articles and overestimating the beneficial effect of the treatment)* |  |
| - Claiming a beneficial effect of the treatment despite statistically non-significant results - Claiming an equivalent beneficial effect of treatment despite statistically non-significant results in superiority RCTs | Delete this spin and use the generic wording, such as:  Treatment A was not more effective on “primary outcome” than the comparator B in patients with … |
| - Claiming safety of the treatment despite adverse events reported in the scientific articles - Claiming a beneficial effect of the treatment despite a small sample size - Claiming a beneficial effect despite lack of a comparator - Focus on P value instead of magnitude of the treatment effect (effect size) - Interpretation of relative risk as absolute risk - Any other type of misleading interpretation not otherwise classified. | Delete this spin; reword and provide the appropriate information when needed. |
|  |  |
| *Misleading extrapolation (i.e., overgeneralization of study results in news stories to different populations, treatments or outcomes that were not assessed in the study)* |  |
| - Extrapolating animal study results to human application - Extrapolating preliminary study results to clinical application - Extrapolating the effect of study outcomes to other outcomes for the disease - Extrapolating the beneficial effect of the study treatment to a different treatment - Extrapolating from the study participants to a larger or different population | Spin in text: *Misleading extrapolation*  Delete the inappropriate extrapolation. |
| - Inappropriate implication for clinical or daily use (i.e., an improper recommendation or advice to use the treatment in clinical practice or daily use not supported by study results) | Delete the statement and clearly report the immediate unavailability in clinical practice. |
| - Any other types of extrapolation not otherwise classified | Delete the inappropriate extrapolation. |
| Spin in author’s/expert/patient’s quote (interview) | Delete the spin in the quote or the quote if needed. |

RCT, randomised controlled trial.

*Haneef R , Lazarus C , Ravaud P , et al. Interpretation of results of studies evaluating an intervention highlighted in google health news: a cross-sectional study of news. PLoS One 2015;10:e0140889
