# Supplementary material for: Three randomized controlled trials evaluating the impact of “spin” in health news stories reporting studies of pharmacologic treatments on patients’/caregivers’ interpretation of treatment benefit
Source: BMC Med. 2019 Jun 4;17:105. doi: 10.1186/s12916-019-1330-9 (PMC6547451; doi:10.1186/s12916-019-1330-9)
Supplement: Supplementary file 6 — Description of spin and modifications performed in the rewritten news stories without spin by study type. (DOCX 21 kb) [file 12916_2019_1330_MOESM6_ESM.docx]

**Additional file 6:** Description of spin and modifications performed in the rewritten news stories without spin by study type

| Spin | Modifications | Pre-clinical study*  n = 10 | Phase I/II non-randomized trial*  n = 10 | Phase III/IV RCT*  n = 10 | Total*  n = 30 |
| --- | --- | --- | --- | --- | --- |
| Misleading information in headline | Delete misleading information and report appropriate information | **10** | **10** | **10** | **30** |
| Misleading reporting (i.e., incomplete or inadequate reporting of any important information in the context of the research that could be misleading for the reader) |  | **10** | **10** | **10** | **30** |
| Misleading reporting of study design | Report the appropriate study design. | 10 | - | - | 10 |
| Not reporting study population if an animal study | Report animal study subjects | 7 | - | - | 7 |
| Selective reporting of outcomes favoring the beneficial effect of the treatment (e.g., statistically significant results for efficacy outcomes or statistically non-significant results for safety outcomes) | Report the results for all primary outcomes. | - | 2 | 4 | 6 |
| Not reporting adverse events | Report adverse events when higher in one group. | 1 | 4 | 7 | 12 |
| Use of linguistic spin (i.e., any word or expression emphasizing the beneficial effect of the treatment) | Delete linguistic spin | 10 | 9 | 10 | 29 |
| Not reporting study limitations  Not reporting any caution about study design and results | Report study limitations and cautions with standardized text as reported in table 1. | 10 | 10 | 10 | 30 |
| Any other type of misleading reporting | Delete spin |  | 1 |  | 1 |
| Misleading interpretation (i.e., interpretation of the study results in news stories that is not consistent with the results reported in the scientific articles and overestimating the beneficial effect of the treatment) |  | **2** | **10** | **7** | **19** |
| Claiming a beneficial effect of treatment despite statistically non-significant results | Delete this spin and use the generic wording, such as:  Treatment A was not more effective on “primary outcome” than the comparator B in patients with … | - | 2 | 4 | 6 |
| Claiming an equivalent beneficial effect of treatment despite statistically non-significant results in superiority RCTs | Delete this spin and use the generic wording, such as:  Treatment A was not more effective on “primary outcome” than the comparator B in patients with … | - | - | - | - |
| Claiming safety of the treatment despite adverse events reported in the scientific articles | Delete this spin; reword and provide the appropriate information when needed. | - | 3 | - | 3 |
| Claiming a beneficial effect of the treatment despite a small sample size not reported | Delete this spin; reword and provide the appropriate information when needed. | - | 3 | - | 3 |
| Claiming a beneficial effect of the treatment despite lack of comparator | Delete this spin; reword and provide the appropriate information when needed. | - | 8 | - | 8 |
| Focus on p-value instead of magnitude of the treatment effect (effect size) | Delete this spin; reword and provide the appropriate information when needed. | - | - | 2 | 2 |
| Any other type of misleading interpretation | Delete the spin, interpret appropriately | 2 | 4 | 2 | 8 |
| Misleading extrapolation (i.e., overgeneralization of study results in news stories to different populations, treatments or outcomes that were not assessed in the study) |  | **10** | **9** | **7** | **26** |
| Extrapolating animal study results to human application | Delete inappropriate extrapolation | 8 | - | - | 8 |
| Extrapolating preliminary study results to clinical application | Delete inappropriate extrapolation | - | 5 | - | 5 |
| Extrapolating the effect of study outcomes to other outcomes for the disease | Delete inappropriate extrapolation | 6 | 5 | 2 | 13 |
| Extrapolating the beneficial effect of the study treatment to a different treatment | Delete inappropriate extrapolation | - | 1 | - | 1 |
| Extrapolating from the study participants to a larger or different population | Delete inappropriate extrapolation | - | 1 | - | 1 |
| Inappropriate implication for clinical or daily use | Delete inappropriate extrapolation | 5 | 2 | 5 | 12 |
| Inappropriate extrapolation from treatment in development to immediately available in clinical practice | Clearly report the immediate unavailability in clinical practice | 10 | 1 | - | 11 |
| Any other types of extrapolation | Delete inappropriate extrapolation | - | 1 | 1 | 2 |
| Spin in author’s/expert/patient’s quote |  |  |  |  |  |
| Spin in a quote from the researcher/investigator of the study or an expert commenting on the study | Delete spin in the quote or delete the quote | 8 | 6 | 4 | 18 |
| Spin in patient’s quote | Delete spin in the quote or delete the quote | - | - | 1 | 1 |

RCT, randomised controlled trial.

*At least one spin was identified and modified
