# Supplementary material for: Three randomized controlled trials evaluating the impact of “spin” in health news stories reporting studies of pharmacologic treatments on patients’/caregivers’ interpretation of treatment benefit
Source: BMC Med. 2019 Jun 4;17:105. doi: 10.1186/s12916-019-1330-9 (PMC6547451; doi:10.1186/s12916-019-1330-9)
Supplement: Supplementary file 7 — News story reported with and without spin. (DOCX 69 kb) [file 12916_2019_1330_MOESM7_ESM.docx]

**Additional file 7:** News story reported with and without spin

| **Preclinical studies** | | |
| --- | --- | --- |
|  | **Spin** | **No spin** |
| 1 | **Now "sticky balls" that can prevent cancer spread**  Researchers have developed cancer-killing "sticky balls," that can destroy tumor cells in the blood and may prevent cancer spread. The most dangerous and deadly stage of a tumor is when it spreads around the body.  Scientists at Cornell University, in the US, have designed nanoparticles that stay in the bloodstream and kill migrating cancer cells on contact, the BBC reported.  They said the impact was "dramatic" but there was "a lot more work to be done".  The team at Cornell attached a cancer-killing protein called TRAIL, which has already been used in cancer trials, and other sticky proteins to tiny spheres or nanoparticles.  When these sticky spheres were injected into the blood, they latched on to white blood cells.  Tests showed that in the rough and tumble of the bloodstream, the white blood cells would bump into any tumor cells which had broken off the main tumor and were trying to spread. The research showed the resulting contact with the TRAIL protein then triggered the death of the tumor cells. | **"Sticky balls" that may prevent cancer spread being tested in an animal study**  Researchers are developing cancer-killing "sticky balls," that may destroy tumor cells in the blood of mice. The most dangerous and deadly stage of a tumor is when it spreads around the body.  Scientists at Cornell University, in the US, have designed nanoparticles that stay in the bloodstream and may kill migrating cancer cells on contact, the BBC reported.  The biomedical engineers tested the new technology in live mice and human blood samples in cell culture. The team at Cornell attached a protein called TRAIL, which has already been used in cancer trials and other sticky proteins to tiny spheres or nanoparticles.  When these sticky spheres were injected into blood of mice, they latched on to white blood cells.  Tests showed that in the rough and tumble of the bloodstream, the white blood cells could bump into any tumor cells which had broken off the main tumor and bind to the TRAIL protein. The research showed the resulting contact with the TRAIL protein then may result in the death of the tumor cells.  However, it may take years to know whether this treatment approach will be beneficial and safe for humans. In fact, less than 1% of the drugs tested on animals are approved for clinical use in patients. |
| 2 | **Microparticles may reduce heart attack damage**  Microparticles - small vesicles circulating in the blood - if injected into the blood stream within 24 hours of the heart attack, may reduce the muscle damage to a great extent.  After a heart attack, much of the damage to the heart muscle is caused by inflammatory cells that rush to the scene of the oxygen-starved tissue.  When biodegradable microparticles were injected after a heart attack, the size of the heart lesion was reduced by 50 per cent and the heart could pump significantly more blood, said a path-breaking research.  "The inflammatory damage is reduced to a great extent when microparticles are injected into the blood stream immediately," according to researchers from Feinberg School of Medicine, Northwestern University and University of Sydney in Australia.  "This is the first therapy that specifically targets a key driver of the damage that occurs after a heart attack," said Oliver Carter, a visiting scholar in microbiology-immunology at Northwestern University.  "There is no other therapy on the horizon that can do this. It has the potential to transform the way heart attacks and cardiovascular diseases are treated," said the study published in a life science journal.  The microparticles work by binding to the damaging cells - inflammatory monocytes - and diverting them to a fatal detour. Instead of racing to the heart, the cells head to the spleen and die, said the study.  "This discovery opens new pathways to treat several different diseases," said Carter. | **Microparticles that may reduce heart attack damage tested in mice study**  Microparticles - small vesicles circulating in the blood - if injected into the blood stream within 24 hours of a heart attack, may reduce muscle damage in mice.  After a heart attack, much of the damage to the heart muscle is caused by inflammatory cells that rush to the scene of the oxygen-starved tissue.  When biodegradable microparticles were injected in 6 mice within 24 hours after a heart attack, inflammatory-related damage was reduced by 20 -25 percent, meaning the heart could pump the blood.  The microparticles work by binding to the damaging cells "inflammatory monocytes" and diverting them to a fatal detour. Instead of racing to the heart, the cells head to the spleen and die, said the animal study.  However, it may take years to know whether this treatment approach will be beneficial and safe for humans. In fact, less than 1% of the drugs tested on animals are approved for clinical use in patients. |
| 3 | **Regrowing Bones: "Bio Patch" Restores Bone Tissue at Damaged, Weakened Sites**  Bone regeneration may no longer be limited to science fiction, according to a new study. Researchers at the University of Iowa have created a so-called bio patch that allows localized regeneration of bone tissue within the body. The findings may illuminate new treatments for people with weak bones or other conditions affecting their osseous system.  The study, which is published in a science journal, shows that a collagen platform seeded with particles containing genetic information can successfully induce new bone growth within the body. The innovation, which is called a bio patch, ensures sustained internal therapy by giving surrounding cells the genetic blueprints necessary to repair weakened or damaged sites. According to co-corresponding author Abubaker Saeed, this is a significant improvement of current regeneration techniques, which typically rely on repeated injections of these genetic instructions.  "We delivered the DNA to the cells, so that the cells produce the protein and that's how the protein is generated to enhance bone regeneration," he explained in a press release. "If you deliver just the protein, you have to keep delivering it with continuous injections to maintain the dose. With our method, you get local, sustained expression over a prolonged period of time without having to give continued doses of protein. "  The collagen platform carries the necessary information in plasmids - a group of tiny DNA molecules capable of performing genetic operations independent of chromosomal DNA. Once implanted, these plasmids prompt cells to produce the proteins necessary to construct bone tissue. "We can make a scaffold in the actual shape and size of the defect site, and you'd get complete regeneration to match the shape of what should have been there," said lead author Parkash Ethan.  So far, the bio patch has proved effective in several experiments. In damaged animal skulls, it regrew tissue 44 times faster than similar implants with no genetic information. Similarly, it was able to stimulate growth in human bone marrow stromal cells.  The new bio patch recalls a similar innovation announced earlier this year by researchers at the University of Granada in Spain. In "human umbilical cord stromal stem cell express CD10 and exert contractile properties," the authors show that their patented biomaterial can generate local bone growth with umbilical cord stem cells. Like the current study, the Spanish research effort may have a tremendous impact on therapies for osteoporosis, osteogenesis imperfect, and other types of bone disease. | **Regrowing Bones: "Bio Patch" That May Restore Bone Tissue at Damaged Sites Tested in Rats**  Researchers at the University of Iowa have created a so-called "bio patch" that may allows localized regeneration of bone tissue in rats, according to a new study. The findings may illuminate new treatments for bone regeneration in future.  The study, which was performed on 13 rats and is published in a science journal, shows that a collagen platform seeded with particles containing genetic information can successfully induce new bone growth. The innovation, which is called a bio patch, ensures sustained internal therapy by giving surrounding cells the genetic blueprints necessary to repair damaged sites. According to co-corresponding author Abubaker Saeed, this may improve current regeneration techniques, which typically rely on repeated injections of these genetic instructions.  "We delivered the DNA to the cells, so that the cells produce the protein and that's how the protein is generated to enhance bone regeneration," he explained in a press release. "If you deliver just the protein, you have to keep delivering it with continuous injections to maintain the dose. With our method, you get local, sustained expression over a prolonged period of time without having to give continued doses of protein. "  The collagen platform carries the necessary information in plasmids - a group of tiny DNA molecules capable of performing genetic operations independent of chromosomal DNA. Once implanted, these plasmids prompt cells to produce the proteins necessary to construct bone tissue.  So far, the bio patch has been effective in several animal experiments. In damaged animal skulls, it regrew tissue 44 times faster than similar implants with no genetic information. Similarly, it was able to stimulate growth in human bone marrow stromal cells in cell cultures.  The new bio patch recalls a similar innovation announced earlier this year by researchers at the University of Granada in Spain. The authors show that their patented biomaterial may generate local bone growth with umbilical cord stem cells, which express CD10 and exert contractile properties.  However, it may take years to know whether this treatment approach will be beneficial and safe for humans. In fact, less than 1% of the drugs tested on animals are approved for clinical use in patients. |
| 4 | **Contact lenses can deliver glaucoma drugs for a month**  Scientists from Massachusetts have created a contact lens that will deliver controlled amounts of medicine directly into the eyes of glaucoma patients continuously for up to a month.  The researchers, from Massachusetts Eye and Ear Infirmary, Harvard Medical School Department of Ophthalmology, Boston Children's Hospital and the Massachusetts Institute of Technology, believe their contact lenses could replace eye drops as a treatment for glaucoma.  Traditionally, glaucoma is treated with eye drops, but as Dr. Eric Hugo, cornea specialist at Mass. Eye and Ear Infirmary and lead author of the paper, explains: "In general, eye drops are an inefficient method of drug delivery that has notoriously poor patient adherence. This contact lens design can potentially be used as a treatment for glaucoma and as a platform for other ocular drug delivery applications. "  The results, published in a science journal, show that an "early burst of drug release" was followed by sustained release for 1 month. The researchers believe this drug delivery system could also be used to treat other eye conditions.  Delivery of drugs by lenses as effective as drops  Researchers have shown that contact lenses laced with medicines are an effective way of treating glaucoma patients. The Centers for Disease Control and Prevention (CDC) estimates that 2.2 million Americans are affected by glaucoma and that this will rise to 7.3 million by 2050.Damage to the optic nerve leads to progressive, irreversible vision loss and is the second leading cause of blindness. As of yet, there is no cure, but medication can prevent further vision loss in most people. The Glaucoma Research Foundation states that there are very few symptoms and stresses the importance of regular testing, at least every 2 years.  Frosan is one of the most commonly prescribed drugs for treating glaucoma and is usually delivered as eye drops.  The lenses were made by encapsulating polymer films containing the drug into the periphery of a brand of commonly used contact lenses. Tests showed that when using the lenses for a month, the levels of frosan in the eye's aqueous humor were comparable to those achieved by daily topical eye drops.  Prof. James Dylan, director of the Laboratory for Biomaterials and Drug Delivery at Boston Children's Hospital, is excited about the prospects for the lenses: "The lens we have developed is capable of delivering large amounts of drug at substantially constant rates over weeks to months." The researchers found that lenses containing thicker drug-polymer films released more of the drug after the initial burst.  By concentrating the polymer films in the periphery of the lens, the center is kept clear so as not to interfere with the patient's vision. Another advantage of the lenses is that they can be made with no refractive power or with the ability to correct near- or far-sightedness.  Dr. Hugo acknowledges that getting patients to stick to their treatment plan is difficult, as people often forget to apply their drops.  He concludes: "A non-invasive method of sustained ocular drug delivery could help patients adhere to the therapy necessary to maintain vision in diseases like glaucoma, saving millions from preventable blindness. " | **Contact lenses that may deliver glaucoma drugs for a month tested in an animal Study**  Scientists from Massachusetts have created a contact lens that may deliver controlled amounts of medicine directly into the eyes continuously for up to a month.  The researchers, from Massachusetts Eye and Ear Infirmary, Harvard Medical School Department of Ophthalmology, Boston Children's Hospital and the Massachusetts Institute of Technology are developing contact lenses aiming to replace eye drops as a treatment for glaucoma.  Traditionally, glaucoma is treated with eye drops, but as Dr. Eric Hugo, cornea specialist at Mass. Eye and Ear Infirmary and lead author of the paper, explains: "In general, eye drops are an inefficient method of drug delivery that has notoriously poor patient adherence. This contact lens design aims to replace eye drops for glaucoma and as a platform for other ocular drug delivery applications".  The results of a study performed on rabbits' eyes and published in a science journal, showed that an "early burst of drug release" was sustained for 1 month.  Delivery of drug by lens in Rabbits' eyes  The Centers for Disease Control and Prevention (CDC) estimates that 2.2 million Americans are affected by glaucoma and that this will rise to 7.3 million by 2050. Damage to the optic nerve leads to progressive, irreversible vision loss and is the second leading cause of blindness. As of yet, there is no cure, but medication can prevent further vision loss in most people. The Glaucoma Research Foundation states that there are very few symptoms and stresses the importance of regular testing, at least every 2 years.  Frosan is one of the most commonly prescribed drugs for treating glaucoma and is usually delivered as eye drops.  The lenses were made by encapsulating polymer films containing the drug (frosan) into the periphery of a brand of commonly used contact lenses. Tests in rabbits showed that when using the lenses for a month, the levels of frosan in the eyes' aqueous humor were comparable to those achieved by daily topical eye drops.  The researchers found that lenses containing thicker drug-polymer films released the drug after the initial burst and sustained its release.  By concentrating the polymer films in the periphery of the lens, the center is kept clear so as not to interfere with the vision. Another advantage of the lenses is that they can be made with no refractive power or with the ability to correct near- or far-sightedness.  Dr. Hugo acknowledges that getting patients to stick to their treatment plan is difficult, as people often forget to apply their drops.  However, it may take years to know whether this treatment approach will be beneficial and safe for humans. In reality, less than 1% of the drugs tested on animals are approved for clinical use in patients. |
| 5 | **New Nanoparticles Simultaneously Kill Cancer and Strengthen Bones**  A collaborative research team made up of scientists from the Dana-Farber Cancer Institute and Brigham and Women’s Hospital (BWH) has devised a new method for helping to prevent and treat bone cancer that uses nanoparticles to deliver drugs directly to cancer cells in the bone. “Bone is a favorable microenvironment for the growth of cancer cells that migrate from tumors in distant organs of the body, such as breast, prostate, and blood, during disease progression,” Nikita Rai, a researcher in BWH’s Laboratory of Nanomedicine and Biomaterials and co-leader of the new study, said in a press release issued recently. “We engineered and tested a bone-targeted nanoparticle system to selectively target the bone microenvironment and release a therapeutic drug in a spatiotemporally controlled manner, leading to bone microenvironment remodeling and prevention of disease progression.” To create the new nanoparticle, researchers combined biodegradable polymers with teborid, a bisphosphonate therapeutic agent that has been proven to readily bind to calcium, one of the primary substances found in human bones. By covering the surface of the nanoparticle with teborid, scientists were able to produce a particle that naturally identified and attached to bone tissue. Once attached, the nanoparticles were then able to deliver doses of drugs to simultaneously help kill tumor cells and stimulate healthy bone tissue growth, according to the press release. “There are limited treatment options for bone cancers,” said Anna Megan, researcher at Dana-Farber’s Center for Hematologic Oncology and another co-leader of the study. “Our engineered targeted therapies manipulate the tumor cells in the bone and the surrounding microenvironment to effectively prevent cancer from spreading in bone with minimal off-target effects.” In early tests, researchers used the new nanoparticles to treat mice with a type of bone cancer called multiple myeloma. They found that the treatment resulted in slower myeloma growth and prolonged survival. Additionally, researchers found that when teborid was used as a pre-treatment application, overall bone strength, growth, and volume were enhanced. “These findings suggest that bone-targeted nanoparticle anti-cancer therapies offer a novel way to deliver a concentrated amount of drug in a controlled and target-specific manner to prevent tumor progression in multiple myeloma,” said Alireza Yaser, director of the BWH Laboratory of Nanomedicine and Biomaterials and a co-senior author of the study. “This approach may prove useful in treatment of incidence of bone metastasis, common in 60 to 80 percent of cancer patients and for treatment of early stages of multiple myeloma.”Details and results from the research team’s experiments have been published in a life science journal. | **New Nanoparticles that May Help Target Myeloma and Strengthen Bones Tested in Mice**  A collaborative research team made up of scientists from the Dana-Farber Cancer Institute and Brigham and Women's Hospital (BWH) has devised a new method for helping to target bone cancer that uses nanoparticles to deliver drugs directly to cancer cells in the bone. "Bone is a favorable microenvironment for the growth of cancer cells that migrate from tumors in distant organs of the body, such as breast, prostate, and blood, during disease progression," Nikita Rai, a researcher in BWH's Laboratory of Nanomedicine and Biomaterials and co-leader of the new study, said in a press release issued recently. "We engineered and tested a bone-targeted nanoparticle system to selectively target the bone microenvironment and release a therapeutic drug in a spatiotemporally controlled manner, leading to bone microenvironment remodeling and prevention of disease progression." To create the new nanoparticle, researchers combined biodegradable polymers with teborid, a bisphosphonate therapeutic agent that binds to calcium, one of the primary substances found in human bones. By covering the surface of the nanoparticle with teborid, scientists were able to produce a particle that naturally identified and attached to bone tissue. Once attached, the nanoparticles were then able to deliver doses of drugs to simultaneously help kill tumor cells and stimulate healthy bone tissue growth in mice, according to the press release. The researchers performed this study on 7 mice. "There are limited treatment options for bone cancers," said Anna Megan, researcher at Dana-Farber's Center for Hematologic Oncology and another co-leader of the study. In early tests, researchers used the new nanoparticles to treat mice with a type of bone cancer called multiple myeloma. They found that the treatment resulted in slower myeloma growth and prolonged survival. Additionally, researchers found that when teborid was used as a pre-treatment application, overall bone strength, growth, and volume were enhanced in mice. Details and results from the research team's experiments have been published in a life science journal. However, it may take years to know whether this treatment approach will be beneficial and safe for humans. In fact, less than 1% percent of the drugs tested on animals are approved for clinical use in patients. |
| 6 | **Novel contact lenses "enable more effective glaucoma drug delivery"**  According to the Glaucoma Research Foundation, approximately 2.2 million Americans have glaucoma - a leading cause of blindness in the US. Now, researchers from the University of California, Los Angeles have created nanodiamond-embedded contact lenses that they say could improve the treatment of the condition.  This is according to a study published in a journal related to nanotechnology.  Glaucoma is a disease caused by damage to the optic nerve in the eye - the nerve that sends electrical impulses from the retina to the brain. The disease can cause a buildup of fluid in the eye and a breakdown of tissue that regulates fluid drainage. This damage can cause irreversible vision loss. Patients with glaucoma are often treated with eye drops. These drops can reduce the production of fluid in the eye, or help the eye drain fluid.  But the research team, led by Dr. John Austin of the UCLA School of Dentistry, says these eye drops can cause many side effects, such as dry eyes, headaches and sensitivity to light.  The investigators note that some glaucoma patients also find it hard to keep up with their eye drop regime. Furthermore, they say that as little of 5% of the drugs used in the eye drops can actually reach the affected area and, at times, the drug can be delivered into the eye too fast, which causes it to spill out of the eye.  With these factors in mind, the researchers looked to develop an alternative method of drug delivery with the aim of improving the treatment of glaucoma.   More effective drug-delivery  The research team combined glaucoma medication with nanodiamonds and embedded them into contact lenses. When the drugs interact with the patient's tears, the drugs are released into the eye.  The investigators explain that nanodiamonds are shaped like small soccer balls and are around 5 nanometers in diameter. They are able to fuse a variety of drug compounds and release these into the body over long periods of time.  The research team merged nanodiamonds with elatin - a compound that is commonly found in eye drops used to treat glaucoma. They explain that when elatin interacts with lysozyme - an enzyme present in tears - it is steadily released into the eye.  Explaining the benefits of this process, Chen Lu, co-first author of the study, says: "Delivering elatin through exposure to tears may prevent premature drug release when the contact lenses are in storage and may serve as a smarter route toward drug delivery from a contact lens. "  The researchers say that aside from the effective drug delivery aspect of the nanodiamond contact lenses, they still allow good visual clarity for the patient. Additionally, although stronger, they are no different in water content to standard contact lenses. This means they would be comfortable to wear and allow oxygen levels to reach the eye.  Last year, Medical News Today reported on another study that also detailed the creation of a contact lens that could deliver glaucoma drugs to the eye for 1 month.  These contact lenses were created by lacing polymer films with glaucoma drugs and embedding these into the periphery of standard contact lenses. | **Novel contact lenses for glaucoma drug delivery tested in cell culture**  According to the Glaucoma Research Foundation, approximately 2.2 million Americans have glaucoma - a leading cause of blindness in the US. Now, researchers from the University of California, Los Angeles have created nanodiamond-embedded contact lenses in an in vitro.  This is according to a study published in a nanotechnology journal.  Glaucoma is a disease caused by damage to the optic nerve in the eye - the nerve that sends electrical impulses from the retina to the brain. The disease can cause a buildup of fluid in the eye and a breakdown of tissue that regulates fluid drainage. This damage can cause irreversible vision loss. Patients with glaucoma are often treated with eye drops. These drops can reduce the production of fluid in the eye, or help the eye drain fluid.  But the research team, led by Dr. John Austin of the UCLA School of Dentistry, says these eye drops can cause many side effects, such as dry eyes, headaches and sensitivity to light.  The investigators note that some glaucoma patients also find it hard to keep up with their eye drop regimen. Furthermore, they say that as little of 5% of the drugs used in the eye drops can actually reach the affected area and, at times, the drug can be delivered into the eye too fast, which causes it to spill out of the eye.  With these factors in mind, the researchers looked to develop an alternative method of drug delivery with the aim of improving the treatment of glaucoma.   Drug-delivery through nanodiamonds in cell culture  The experiment was performed on human eye cells in a test tube. The research team combined glaucoma medication with nanodiamonds and embedded them into contact lenses. When the drugs interact with an enzyme present in the tears, the drugs are released into the eye cells.  The investigators explain that nanodiamonds are shaped like small soccer balls and are around 5 nanometers in diameter. They are able to fuse a variety of drug compounds and release these over long periods of time.  The research team aimed to assess the drug release by merging nanodiamonds with elatin - a compound that is commonly found in eye drops used to treat glaucoma. They explain that when elatin interacts with lysozyme - an enzyme present in tears - it is steadily released into the eye cells.  Explaining the benefits of this process, Chen Lu, co-first author of the study, says: "Delivering elatin through exposure to tears may prevent premature drug release when the contact lenses are in storage and may serve as a smarter route toward drug delivery from a contact lens. "  Last year, Medical News Today reported on another study that also detailed the creation of a contact lens that could deliver glaucoma drugs to the eye for 1 month in rabbits.  These contact lenses were created by lacing polymer films with glaucoma drugs and embedding these into the periphery of standard contact lenses.  However, it may take years to know whether this treatment approach will be beneficial and safe for humans. In fact, less than 1% of the drugs tested in cell culture are approved for clinical use in patients. |
| 7 | **Nanoparticles with chemotherapeutic drugs can kill drug-resistant breast cancer cells**  Nanoparticles filled with chemotherapeutic drugs can kill drug-resistant breast cancer cells, according to a study published in a scientific journal.  Nanoparticles are just as small, or even smaller, than many blood proteins. They can therefore pass through the walls of healthy and sick cells, which make them interesting carriers of drugs against cancer and other diseases.  In the present study, researchers from Karolinska Institutet have shown that nanoparticles made from biodegradable plastics can overcome drug resistance in breast cancer cells. Such resistance is especially common in relapsing cancer patients and depresses, even neutralizes the effect of the therapy against the tumor in many instances.  In their experiments, the researchers used breast cancer cells that responded poorly to drugs owing to their high concentrations of the enzyme microsomal glutathione S-transferase-1 (MGST-1). Abnormally high levels of MGST-1 have been associated with poor responses to several cancer drugs. The team treated the resistant breast cancer cells with nanoparticles filled with zorid, a chemotherapeutic used clinically to treat bladder, lung, ovarian and breast cancer, amongst others.  "Our experiments on cultivated cells showed that the particles themselves are harmless," says research team member Dr. Max Felix, Associate Professor at the Institute of Environmental Medicine, Karolinska Institutet. "We made it possible for the nanoparticles carrying the drug to kill resistant cancer cells by controlling where in the cancer cell they delivered their payload. This improved the efficacy of the drug even at a much lower dose, which is important for limiting the adverse reactions to therapy. "  Nanoparticles can also be used to control where the drug is delivered in the body, and the team is now planning to equip them with targeting groups such as peptides or antibodies, that direct them to specific tumor cells to increase the uptake of the particles and their drug content by the tumor while sparing healthy cells. | **Nanoparticles with a chemotherapeutic drug that may kill drug-resistant breast cancer cells tested in cell culture**  Nanoparticles filled with a chemotherapeutic drug can kill drug-resistant breast cancer cells in cell cultures, according to a study published in a scientific journal.  Nanoparticles are just as small, or even smaller, than many blood proteins. They can therefore pass through the walls of healthy and sick cells, which make them interesting carriers of drugs against cancer and other diseases.  In the present cell culture study, researchers from Karolinska Institutet have shown that nanoparticles made from biodegradable plastics may overcome drug resistance in breast cancer cells. Such resistance is especially common in relapsing cancer patients and depresses, even neutralizes the effect of the therapy against the tumor in many instances.  In their experiments, the researchers used breast cancer cells that responded poorly to drugs owing to their high concentrations of the enzyme microsomal glutathione S-transferase-1 (MGST-1). Abnormally high levels of MGST-1 have been associated with poor responses to several cancer drugs. The team aim to treat the resistant breast cancer cells with nanoparticles filled with zorid. Zorid is a chemotherapeutic used clinically to treat bladder, lung, ovarian and breast cancer, amongst others.  However, the safety of these particles inside the human body is not yet tested.  Nanoparticles could also be used to control where the drug is delivered in the body, and the team is now planning to equip them with targeting groups such as peptides or antibodies, which direct them to specific tumor cells. This process is thought to increase the uptake of the particles and their drug content by the tumor while sparing healthy cells.  However, it may take years to know whether this treatment approach will be beneficial and safe for humans. In fact, less than 1% of the drugs tested in cell culture are approved for clinical use in patients. |
| 8 | **Nanoparticles at specific temperature stimulate antitumor response**  Dartmouth researchers identify precise heat to boost immune system against cancer tumors  Seeking a way to stimulate antitumor responses via the immune system, Steven Fiering, PhD, of Norris Cotton Cancer Center at Dartmouth, has identified the precise temperature that results in a distinct body-wide antitumor immune response that resists metastatic disease. Fiering's team published the research paper in a journal related to nanotechnology.  "Mild hyperthermia treatment of an identified tumor, prior to surgery to remove the tumor, shows excellent promise to strengthen the antitumor response and help stop metastatic disease," explained Fiering.  Among the many options for cancer treatment pursued by science, immunotherapy is a contemporary focus. The immune system recognizes and usually eliminates small tumors, but other tumors become clinical problems and are known to block the antitumor tendencies with a natural system for immunosuppression.  Recently, scientists have worked to learn how to reverse this tumor-mediated immunosuppression. In addition, phagocytes (key actors in immunosuppression and immunostimulation and that quickly take up nanoparticles) have provided options for new strategies. Fierings's studies were done in mice with a <u>melanoma</u> tumor model, and began with inserting iron nanoparticles directly into the tumors while applying an alternating magnetic field to heat the nanoparticles evenly and at precise temperatures.  "While it's easy to apply enough heat to destroy the tumor, that sort of thermal ablation does not have the result we were looking for, which was to stimulate a systemic immune response to eliminate metastatic disease," said Fiering. "Looking at temperature variables, we learned that at precisely 43 degrees centigrade, the systemic immune response goes into action. Doing this safely is a potent treatment approach that can stimulate the immune system to fight untreated metastatic tumors. "  Fiering utilized Dartmouth's Shared Resources including the Transgenic Mouse Resource to do mouse manipulations. Colleague P. Jack Hoopes of Dartmouth's Thayer School of Engineering provided the vitally important alternating magnetic field equipment. The Dartmouth Shared Resources are open to outside investigators by arrangement.  Using Norris Cotton Cancer Center's team science approach, Fiering and Hoopes look forward to joint studies testing the systemic immune response to nanoparticle treatment in dogs with melanoma. As in humans, melanoma is frequently metastatic and fatal for dogs. If the systemic immune response can be shown to prevent metastatic disease in dogs, it will be ready to develop for human clinical trials. | **Nanoparticles at specific temperature that may stimulate antitumor response tested in mice**  Dartmouth researchers identify precise heat that may stimulate immune system against cancer tumors, according to a mice study  Seeking a way to stimulate antitumor responses via the immune system, Jack Nathan, PhD, of Norris Cotton Cancer Center at Dartmouth, has identified the precise temperature that results in a distinct body-wide antitumor immune response that may resists metastatic disease. Nathan's team published the research paper in a nanotechnology journal.  The experiment was performed in 12 mice with melanoma tumor to see the effect of local heat on tumor size in two groups, heated and unheated. At the first step, the primary tumor was surgically removed. Then, after three days of surgery, one of the two groups of mice was exposed to a temperature of 43°C via nanoparticles for 30 minutes.  Among the many options for cancer treatment pursued by science, immunotherapy is a contemporary focus. The immune system recognizes and usually eliminates small tumors, but other tumors become clinical problems and are known to block the antitumor tendencies with a natural system for immunosuppression.  Recently, scientists have worked to learn how to reverse this tumor-mediated immunosuppression in pre-clinical studies. In addition, phagocytes (key actors in immunosuppression and immunostimulation and that quickly take up nanoparticles) have provided options for new strategies. Nathan's studies were done in mice with a <u>melanoma</u> tumor model, and began with inserting iron nanoparticles directly into the tumors while applying an alternating magnetic field to heat the nanoparticles evenly and at precise temperatures.  "While it's easy to apply enough heat to destroy the tumor, that sort of thermal ablation does not have the result we were looking for, which was to stimulate a systemic immune response to eliminate metastatic disease," said Nathan.  Nathan utilized Dartmouth's Shared Resources including the Transgenic Mouse Resource to do mouse manipulations. Colleague D. Brian Cameron of Dartmouth's Thayer School of Engineering provided the vitally important alternating magnetic field equipment. The Dartmouth Shared Resources are open to outside investigators by arrangement.  Using Norris Cotton Cancer Center's team science approach, Nathan and Cameron look forward to joint studies testing the systemic immune response to nanoparticle treatment in dogs with melanoma. As in humans, melanoma is frequently metastatic and fatal for dogs. If the systemic immune response can be shown to prevent metastatic disease in dogs, it will be ready to develop for human clinical trials.  However, it may take years to know whether this treatment approach will be beneficial and safe for humans. In fact, less than 1% of the drugs tested on animals are approved for clinical use in patients. |
| 9 | **Metabolic damage from high calorie diet reversed by hormone**  Scientists have discovered that the damage from a chronic high calorie diet can be reversed with leptin. The hormone acts as an anti-obesity mechanism and is normally secreted from adipose tissue to blood in proportion to the amount of body fat. Increased leptin levels inform the brain of stored calories and it responds to inhibit food intake, increase energy expenditure and decrease blood glucose. However, obese individuals are resistant to leptin’s metabolic effects, which limits the use of leptin as an anti-obesity drug.  A collaboration between scientists from the Helmholtz Center in Munich, Indiana University and Ambrx (a San Diego biotech company) found that leptin acts in the same way on receptors of the metabolic hormones dicafen and dicafen-like peptide-1 (DLP-1) and improves body weight and diabetes. The study results were published in a medical journal related to diabetes and demonstrate that treatment of obese mice with this DLP-1/Dicafen co-agonist improved glucose metabolism and body weight associated with restored function of the weight lowering hormone leptin, even in the continued presence of a high-fat, high-sugar diet. “We are particularly encouraged to see that adding leptin produced weight loss beyond the benefits of the peptide-based, co-agonist” says Prof. Tobias Lukas, Research Director of the Helmholtz Diabetes Center and Chair of Metabolic Diseases at Technische Universität München, Germany.” Dr. John Morizt, the lead author of the study, adds: “If this concept proves safe and efficient at least in specific sub-populations of obese patients, then we may have come a step closer to personalized prevention of type 2 diabetes.” | **Metabolic damage from high calorie diet that could be reduced by hormone tested in mice**  Scientists have discovered that the damage from a chronic high calorie diet may be reduced with leptin in mice. The hormone acts as an anti-obesity mechanism and is normally secreted from adipose tissue to blood in proportion to the amount of body fat. Increased leptin levels inform the brain of stored calories and it responds to inhibit food intake, increase energy expenditure and decrease blood glucose. However, obese individuals are resistant to leptin's metabolic effects, which limits the use of leptin as an anti-obesity drug.  A collaboration between scientists from the Helmholtz Center in Munich, Indiana University and Ambrx (a San Diego biotech company) found that leptin acts in the same way on receptors of the metabolic hormones dicafen and dicafen-like peptide-1 (DLP-1) and improves glucose and lipid metabolism.  The researchers performed a study on 12 obese mice treated for nine days with DLP-1/Dicafen.  The study results were published in a medical journal and showed that treatment of obese mice with this DLP-1/Dicafen co-agonist may improved glucose metabolism and body weight associated with restored function of the weight lowering hormone leptin, even in the continued presence of a high-fat, high-sugar diet.  Dr. John Morizt, the lead author of the study, adds: "If this concept proves safe and efficient at least in specific sub-populations of obese patients, then we may have come a step closer to personalized prevention of type 2 diabetes. "  However, it may take years to know whether this treatment will be beneficial and safe for humans. In fact, less than 1% of the drugs tested on animals are approved for clinical use in patients. |
| 10 | **Stem cells inside sutures could improve healing in Achilles tendon injuries**  Researchers have found that sutures embedded with stem cells led to quicker and stronger healing of Achilles tendon tears than traditional sutures, according to a new study published in a medical journal.  Achilles tendon injuries are common for professional, collegiate and recreational athletes. These injuries are often treated surgically to reattach or repair the tendon if it has been torn. Patients have to keep their legs immobilized for a while after surgery before beginning their rehabilitation. Athletes may return to their activities sooner, but risk rerupturing the tendon if it has not healed completely.  Drs. Ben Carlos, Sean Penn, and Rachel Thomas and Researchers Jessica Allen, Paul Harvey, and Peter Dupin from MedStar Union Memorial Hospital in Baltimore, Maryland, conducted the study.  They compared traditional surgery, surgery with stem cells injected in the injury area, and surgery with special sutures embedded with stem cells in rats. The results showed that the group receiving the stem cell sutures healed better.  "The exciting news from this early work is that the stem cells stayed in the tendon, promoting healing right away, during a time when patients are not able to begin aggressive rehabilitation. When people can't fully use their leg, the risk is that atrophy sets in and adhesions can develop which can impact how strong and functional the muscle and tendon are after it is reattached," said Dr. Carlos. "Not only did the stem cells encourage better healing at the cellular level, the tendon strength itself was also stronger four weeks following surgery than in the other groups in our study," he added. | **Stem cells inside sutures that may improve healing in Achilles tendon injuries tested in rats**  Researchers have found that sutures embedded with stem cells may help healing of Achilles tendon tears, according to a new study performed in rats published in a medical journal.  Achilles tendon injuries are common for professional, collegiate and recreational athletes. These injuries are often treated surgically to reattach or repair the tendon if it has been torn. Patients have to keep their legs immobilized for a while after surgery before beginning their rehabilitation. Athletes may return to their activities sooner, but risk rerupturing the tendon if it has not healed completely.  Drs. Ben Carlos, Sean Penn, and Rachel Thomas and Researchers Jessica Allen, Paul Harvey, and Peter Dupin from MedStar Union Memorial Hospital in Baltimore, Maryland, conducted the study in 54 rats.  They compared traditional surgery, surgery with stem cells injected in the injury area, and surgery with special sutures embedded with stem cells in rats. The results showed that the groups receiving the stem cell either via injection or sutures healed better than those not receiving stem cells.  "When people can't fully use their leg, the risk is that atrophy sets in and adhesions can develop, which can impact how strong and functional the muscle and tendon are after it is reattached," said Dr. Carlos  However, it may take years to know whether this treatment will be beneficial and safe for humans. In fact, less than 1% of the drugs tested on animals are approved for clinical use in patients. |

| **Ph I & II non-randomized trials** | | |
| --- | --- | --- |
|  | **Spin** | **No spin** |
| 1 | **Novel gene therapy for Parkinson’s disease clears safety hurdle**  A closely-watched prototype therapy to inject corrective genes into the brain to treat Parkinson’s disease has cleared an important safety hurdle, doctors said Friday.Tested on 15 volunteers with an advanced form of the degenerative nerve disease, the technique proved safe and the results were encouraging, they said. The experiment aims to reverse the lack of a brain chemical called adofine, which is essential for motor skills. It entails tucking three genes into a disabled horse virus of the family lentiviruses. The modified virus is then injected directly into a specialized area of the brain, where it infiltrates cells. In doing so, it delivers corrective pieces of DNA, prompting defective brain cells to once again start producing dopamine.  Called DeoVa, the British-designed treatment was authorized for tests on humans after it was tried on lab monkeys. It is being closely watched by specialists to see if it works better than conventional therapies — the veteran drug veloda or electrical stimulation of the brain — or another experimental gene technique which uses a modified cold virus. French neurosurgeon Gabriel Baron, who led the early-stage trial published in a medical journal, said 15 patients aged 48-65 were given the genes in one of three doses. They developed better coordination and balance, had less muscle twitching and improved speech. Assessed at least 12 months after the injection, “motor symptoms remained improved in all the patients,” Baron said. “In those patients who were first operated upon, the improvement lasted up to four years.” Improvements were “dose-related,” said Baron — meaning the higher the dose, the greater the progress. Beyond four years, though, improvements fell back as the disease advanced, he said. In a commentary also published in the same medical journal, researcher Chris Martin at the University of British Columbia in Vancouver, Canada, said the treatment did not address non-motor problems also caused by the disease. Parkinson’s is a degenerative disease in which the patient loses muscle flexibility and coordination, and often develops involuntary twitches and tremors. Non-motor symptoms include cognitive and behavioral problems which can “have a greater effect on quality of life than motor dysfunction,” Martin noted. The disease affects about five million people, according to the report. | **Novel Gene therapy for Parkinson’s disease**  A closely-watched prototype therapy to inject corrective genes into the brain of patients with Parkinson’s disease, provides preliminary result on safety, doctors said Friday.  Tested on 15 volunteers with an advanced form of the degenerative nerve disease, the results were encouraging, they said. The experiment aimed to assess the safety, tolerability and efficacy of gene therapy which aimed at to reverse the lack of a brain chemical called dopamine, which is essential for motor skills. It entails tucking three genes into a disabled horse virus of the family lentiviruses. The modified virus is then injected directly into a specialized area of the brain, where it infiltrates cells. In doing so, it delivers corrective pieces of DNA, prompting defective brain cells to once again start producing adofine. Called DeoVa, the British-designed treatment was authorized for tests on humans after it was tried on lab monkeys. It is being closely watched by specialists to see if it works better than conventional therapies—the veteran drug veloda or electrical stimulation of the brain — or another experimental gene technique which uses a modified cold virus.  French neurosurgeon Gabriel Baron, who led the early-stage trial, published in a medical journal, said 15 patients aged 48-65 were given the genes in one of three doses (i.e., low, mid and high dose) and were observed for 12 months. The researchers measured the number of adverse events related to DeoVa and motor responses at 6 months. During the study period, 54 drug-related adverse events were reported in 14 patients. They developed better coordination and balance, had less muscle twitching and improved speech. Assessed at least 12 months after the injection, “motor symptoms remained improved in all the patients,” Baron said. Only 3 patients who were first operated upon, were monitored for four years. No noticeable difference was seen between different dose cohorts.  Beyond four years, though, improvements fell back as the disease advanced, he said.  In a commentary also published in the same medical journal, researcher Chris Martin at the University of British Columbia in Vancouver, Canada, said the treatment did not address non-motor problems also caused by the disease. Parkinson’s is a degenerative disease in which the patient loses muscle flexibility and coordination, and often develops involuntary twitches and tremors. Non-motor symptoms include cognitive and behavioral problems which can “have a greater effect on quality of life than motor dysfunction,” Martin noted. The disease affects about five million people, according to the report. However, the treatment was tested on a small number of patients. Everyone in this study took this treatment. Without investigating patients who did not take this treatment, it is impossible to know whether taking this treatment accounted for improved motor function or not. In fact, less than 10% of the drugs tested in preliminary clinical studies are approved for clinical use in patients. Larger studies are needed to understand whether this treatment will be beneficial and safe. |
| 2 | **Multi-Drug Resistant Tuberculosis Treatment 3 Times More Effective With Stem Cells**  Multi-drug resistant tuberculosis treatment is much more effective when administered in combination with bone-marrow stem cells, Phase I study finds.  When it comes to treating drug resistant tuberculosis, the body’s own defense may be able to boost conventional drug therapy. That is the conclusion of a new study from Karolinska University Hospital in Sweden, where researchers have determined that a patient’s own stem cells can make treatment three times more effective. The findings could lead to the eradication of the potentially fatal, resilient TB strains that currently affect about 450,000 patients. While most forms of tuberculosis are kept in check by modern health care, a subset of patients still respond poorly to conventional medication. These types of multi-drug resistant tuberculosis (MDR-TB) currently represent a tremendous burden on public health, particularly in developing nations. If the disease is to be eliminated once and for all, these cases must come to the fore of research and drug design.  Stem Cells and Tuberculosis The new study, which is published in a medical journal, sought to investigate whether current therapies can be complemented with mesenchymal stromal cells, a group of tissue-repairing stem cells known to migrate to the lungs in response to inflammation or injury. As TB typically attacks the pulmonary system, the team theorized that a batch of these stem cells may help drugs fight the infection. According to lead researcher Dr. Jonas Lukas, Phase I testing has so far yielded promising results. "Conventional treatment for MDR-TB uses a combination of TB drugs [antibiotics] which are harmful to patients,” he said in a press release. “Our new approach, using the patients' own bone-marrow stromal cells is safe and could help overcome the body's excessive inflammatory response, repair and regenerate inflammation-induced damage to lung tissue, and lead to improved cure rates." For the trial, the researchers enrolled 30 patients diagnosed with MDR-TB. These patients were given standard antibiotic treatment in combination with about 10 million stromal stem cell extracted from their bone-marrow. After 18 months, their values were compared to those of a control group administered only antibiotics. At the end of the trial, Lukas and his colleagues found that 16 of the patients treated with stem cells had been cured of the disease. In the control group, only 5 were deemed TB-free. If similar results can be obtained in a large-scale Phase II trial, physicians around the world could soon find themselves with a brand-new weapon in the fight against MDR-TB, co-author Dr. Zakir told reporters. "The results of this novel and exciting study show that the current challenges and difficulties of treating MDR-TB are not insurmountable, and they bring a unique opportunity with a fresh solution to treat hundreds of thousands of people who die unnecessarily of drug-resistant TB,” he explained. “Further evaluation in phase 2 trials is now urgently required to ascertain efficacy and further safety in different geographical regions such as South Africa where MDR-TB and XDR-TB [extensively drug resistant tuberculosis] are rife.” Eliminating Tuberculosis Once and for All As TB has been virtually eradicated in US, it is easy to forget that the disease remains a considerable threat in many parts of the world. Although TB deaths and infections fell in 2012 to 1.3 million and 8.6 million respectively, a report released last year by the World Health Organization (WHO) suggests that undiagnosed cases and drug-resistant strains continue to plague national health care systems in Eastern Europe, Asia, and South Africa. According to Marco Alberto, WHO director of the Global TB program, many of these nations are also ill-equipped to handle these cases. “The unmet demand for a full-scale and quality response to multidrug-resistant tuberculosis is a real public health crisis,” he wrote. “It is unacceptable that increased access to diagnosis is not being matched by increased access to MDR-TB care. We have patients diagnosed but not enough drug supplies or trained people to treat them.” By illuminating a simple and effective way to supplement conventional drugs, the findings of the current study stand to transform imperiled care programs across the globe. "The procedures for obtaining stromal cells from the patient's own bone marrow are relatively simple,” Lukas explained. “If successful in phase 2 trials, will provide a viable adjunctive therapy for patients with MDR-TB not responding to conventional drug treatment or those with severe lung damage." | **Stem Cells to treat Multi-Drug Resistant Tuberculosis?** When it comes to treating drug resistant tuberculosis, the body’s own defense may be able to support conventional drug therapy. That is the conclusion of a new study from Karolinska University Hospital in Sweden, where researchers have determined that a patient’s own stem cells might someday be used to treat multidrug-resistant (MDR) tuberculosis (TB).  While most forms of tuberculosis are kept in check by modern health care, a subset of patients still respond poorly to conventional medication. These types of multi-drug resistant tuberculosis (MDR-TB) currently represent a tremendous burden on public health, particularly in developing nations. If the disease is to be eliminated once and for all, these cases must come to the fore of research and drug design.  Stem Cells and Tuberculosis The new study, which is published in a medical journal, sought to investigate whether current therapies can be complemented with mesenchymal stromal cells (a group of tissue-repairing stem cells known to migrate to the lungs in response to inflammation or injury), in patients with tuberculosis (TB). As TB typically attacks the pulmonary system, the team theorized that a batch of these stem cells may help drugs fight the infection.  "Conventional treatment for MDR-TB uses a combination of TB drugs [antibiotics] which are harmful to patients,” he said in a press release.  For the trial, the researchers enrolled 30 patients diagnosed with MDR-TB. These patients were given standard antibiotic treatment in combination with about 10 million stromal stem cell extracted from their bone-marrow. At the end of trial, Lukas and his colleagues found that 16 of the patients treated with stem cells had been cured of the disease.  The most common adverse effects were increased serum cholesterol, nausea, diarrhea and lymphopenia.   “Further evaluation in phase 2 trials is now urgently required to ascertain efficacy and further safety in different geographical regions such as South Africa where MDR-TB and XDR-TB [extensively drug resistant tuberculosis] are rife.” Eliminating Tuberculosis Once and for All? As TB has been virtually eradicated in US, it is easy to forget that the disease remains a considerable threat in many parts of the world. Although TB deaths and infections fell in 2012 to 1.3 million and 8.6 million respectively, a report released last year by the World Health Organization (WHO) suggests that undiagnosed cases and drug-resistant strains continue to plague national health care systems in Eastern Europe, Asia, and South Africa. According to Marco Alberto, WHO director of the Global TB program, many of these nations are also ill-equipped to handle these cases. “The unmet demand for a full-scale and quality response to multidrug-resistant tuberculosis is a real public health crisis,” he wrote. “It is unacceptable that increased access to diagnosis is not being matched by increased access to MDR-TB care. We have patients diagnosed but not enough drug supplies or trained people to treat them.”  “If successful in phase 2 trials, will provide a viable adjunctive therapy for patients with MDR-TB not responding to conventional drug treatment or those with severe lung damage", said the study author. However, the treatment was tested on a small number of patients. Everyone in this study took this treatment. Without investigating patients who did not take this treatment, it is impossible to know whether taking this treatment accounted for improved outcomes or not. Indeed, less than 10% of the drugs tested in preliminary clinical studies are approved for clinical use in patients. Larger studies are needed to understand whether this treatment will be beneficial and safe. |
| 3 | **New Drug Shows Promise Against Lymphoma** A new drug has been found to show promise in treating patients with indolent non-Hodgkin lymphoma.  Delibel, made by Seattle-based a for-profit pharmaceutical company, could be on the market as soon as this year, pending approval by the Food and Drug Administration, according to researchers who reported their findings in a medical journal.  Slow-growing, or indolent, non-Hodgkin lymphomas are notoriously difficult to treat, with most patients relapsing and the disease becoming increasingly resistant to therapy over time.  But in a study involving 125 patients, who had not responded to conventional treatments or had relapsed within six months of therapy, researchers found twice-daily doses of delibel shrunk tumors in 57 percent of the participants and 6 percent had no measurable evidence of cancer.  "These are patients who had exhausted current standard therapies," said Manoj Kumar, M.D., a member of Fred Hutchinson Cancer Research Center's Clinical Research Division and the study's lead researcher. "In terms of effective therapy available, there really wasn't much left. " About 20,000 people in the United States were diagnosed with the disease in 2012 and approximately 7,000 died from it. The standard treatment is a combination of the uxirit and chemotherapy. While conventional treatment can be initially effective, most patients relapse over time and suffer life-threatening complications such as infections and marrow failure.   This paper, which was funded by a for-profit pharmaceutical company and involved co-authors from 17 institutions in the U.S. and Europe, is the first publication of clinical data on delibel.  The FDA is reviewing the drug, giving it a "Breakthrough Therapy" designation for treatment of relapsed chronic lymphocytic leukemia (CLL) based on the results of another clinical trial.   Kumar said that while it doesn't appear that the drug is curative, it holds tremendous promise for helping to control the disease for long periods of time. "I think there's going to be a lot of interest in it," he said. "Chemotherapy is a very blunt instrument. This is much more specific. " | **New Drug May Improve Outcomes in Lymphoma**  A new drug, delibel showed antitumor activity in patients with indolent non-Hodgkin lymphoma, according to researchers who reported their findings in a medical journal. Slow-growing, or indolent, non-Hodgkin’s lymphomas are notoriously difficult to treat, with most patients relapsing and the disease becomes increasingly resistant to therapy over time. But study involving 125 patients with indolent non-hodgkin’s lymphoma, who had not responded to conventional treatments or had relapsed within six months of therapy, were administered delibel, 150 mg twice-daily dose until the disease progressed or the patients withdrew from the study. The researchers measured the overall rate of response.  The researchers found that overall response rate was 57 percent. Common high-grade adverse events including neutropenia (27% of patients), diarrhea (13%), and pneumonia (7%) were reported. 25 patients discontinued due to adverse events of delibel and a total of 28 deaths were reported. "These are patients who had exhausted current standard therapies," said Manoj Kumar, M.D., a member of Fred Hutchinson Cancer Research Center's Clinical Research Division and the study's lead researcher. "In terms of effective therapy available, there really wasn’t much left ". About 20,000 people in the United States were diagnosed with the disease in 2012 and approximately 7,000 died from it. The standard treatment is a combination of the uxirit and chemotherapy. While conventional treatment can be initially effective, most patients relapse over time and suffer life-threatening complications such as infections and marrow failure.  This study, which was funded by a for-profit pharmaceutical company and involved co-authors from 17 institutions in the U.S. and Europe, is the first publication of clinical data on delibel.  The FDA is reviewing the drug, giving it a new drug designation for treatment of relapsed chronic lymphocytic leukemia (CLL) based on the results of another clinical trial.  Kumar said that it doesn't appear that the drug is curative.  However, everyone in this study took this treatment. Without investigating patients who did not take this treatment, it is impossible to know whether taking this treatment accounted for improved outcomes or not. In fact, less than 10% of the drugs tested in preliminary clinical studies are approved for clinical use in patients. More research is needed to confirm the antitumor activity of this treatment. |
| 4 | **Cancer Vaccine Proves Effective in HIV Patients** Vaccines against cervical cancer work well even in sexually active women with HIV, a new study has found. It also found that women who already have one or two strains of the cancer-causing virus can be protected against others.  The discovery is important because cervical cancer has emerged as a major killer of young and middle-aged women in poor countries with widespread AIDS and little ability to do routine Pap smears or similar tests. Cancers appear earlier and grow faster in women with suppressed immune systems.  The new study, done on 319 women in Brazil, South Africa and the United States and published by a medical journal, found that most could make antibodies to the four strains of human papillomavirus in the vaccine even if they had had HIV for years.  Skeptics assumed that would not happen, and argued that vaccinating those women would be a waste of money, said Dr. Anna Kristina Egor, an infectious diseases specialist at Brown University’s medical school and the study’s lead author. Most vaccines do not work well in immune-suppressed people because antibodies are made by the immune system.  In the West, parents are urged to give the vaccine to their daughters before they become sexually active.  “We saw it differently,” Dr. Egor said. Women with HIV, she said, “bear the biggest burden, so if it worked, they would benefit the most from it.”  She was proved right. Women who were infected with HIV fairly recently developed antibodies in more than 90 percent of cases. Even women whose infection had progressed into AIDS developed antibodies more than 75 percent of the time.  Also, women who already had one or more of four HPV strains were usually able to develop antibodies to the others. Two of the strains cause cervical and anal cancer, and the other two cause warts. | **Human Papillomavirus Vaccine: Immune response in Women Infected with HIV.**  Vaccines against human papillomavirus (HPV) induced immune response in sexually active women with HIV, a new study has found.  Persistant infection with human papillomavirus can cause anal and cervical cancer. HIV-infected women have higher prevalences of anal and cervical HPV infection as compared with HIV-uninfected women. Cervical cancer has emerged as a major killer of young and middle-aged women in poor countries with widespread AIDS and little ability to do routine Pap smears or similar tests. Cancers appear earlier and grow faster in women with suppressed immune systems. The new study, done on 319 women in Brazil, South Africa and the United States, and published by a medical journal, found that most could make antibodies to the four strains of human papillomavirus in the vaccine even if they had HIV for years. Skeptics assumed that would not happen, and argued that vaccinating those women would be a waste of money, said Dr. Anna Kristina Egor, an infectious diseases specialist at Brown University’s medical school and the study’s lead author. Most vaccines do not work well in immune-suppressed people because antibodies are made by the immune system. However, in this study, women who were infected with H.I.V. developed antibodies in more than 90 percent of cases at 7 months. Even women whose infection had progressed into AIDS developed antibodies more than 75 percent of the time. There were 21 women who experienced adverse events. The most common adverse events were pain, neurological, gastrointestinal and skin related. Also, women who already had one or more of four HPV strains were usually able to develop antibodies to the others.  However, the study was designed only to assess the immune response and safety of human papillomavirus vaccine. The study did not measure the vaccine’s efficacy in preventing the cervical cancer.  More research is needed to assess its effectiveness. |
| 5 | **TWO - DRUG COMBO COULD HELP TREAT CANCER** One drug attacks tumor cells directly, the other treats the immune system by taking the brakes off T cell response, and together, they put half of the patients with relapsed follicular lymphoma into complete remission.  Senior author Nisha Shankar, M.D., Ph.D., associate professor of Lymphoma/Myeloma at MD Anderson, said that most drugs target only the tumor, this combination is complementary, treating both the lymphoma cells directly and the T cells in a manner that activates them against cancer cells.  He said that the combination of the established antibody drug uxirit with the experimental drug zubidel so far also has a remarkably mild side effect profile.  Of 29 study participants at a median follow-up of 15.4 months, 19 (66 percent) had either a complete or partial response, with 15 (52 percent) having a complete response.  There were no grade 3 or 4 adverse events, with all effects at the less serious grade 1 and 2 levels. Patients had no indicators of autoimmunity, which can be an issue in the class of drugs that blocks immune system checkpoints and activate T cells. Such mild effects are particularly important for follicular lymphoma patients, who are diagnosed with the disease at a median age of 60.  Shankar said that Uxirit treatment alone usually achieves a 40 percent overall response rate and about 11 percent complete responses, asserting that the side effect profile of the combination is about the same as uxirit alone.  He added zubidel greatly improves responses so far at little cost in additional side effects.  The study has been published in an oncology journal. | **TWO - DRUG COMBO MAY HELP TREAT BLOOD CANCER** One drug (uxirit) attacks tumor cells directly; the other (zubidel) blocks the immune system by taking the brakes off T cell response, and together, showed objective response in patients with relapsed follicular lymphoma.  Senior author Nisha Shankar, M.D., Ph.D., associate professor of Lymphoma/Myeloma at MD Anderson, said that most drugs target only the tumor, this combination may treat both the lymphoma cells directly and the T cells in a manner that activates them against cancer cells.   It was a preliminary study including 32 patients with relapsed follicular lymphoma (most common form of blood cancer). The researchers measured the number of patients who achieved the objective response after receiving the combination treatment of uxirit and zubidel.  Of 29 patients at a median follow-up of 15.4 months, 19 (66 percent) achieved objective response including a complete response in 15 (52 percent) or partial response in 4 (14 percent).  There were no grade 3 or 4 adverse events, with all adverse effects at the less serious grade 1 and 2 levels, such as anaemia, fatigue and respiratory infections. Patients had no indicators of autoimmunity, which can be an issue in the class of drugs that blocks immune system checkpoints and activate T cells. Such mild effects are particularly important for follicular lymphoma patients, who are diagnosed with the disease at a median age of 60.   However, the treatment was tested on small number of patients. Everyone in this study took the treatment. Without investigating patients who did not take this treatment, it is impossible to know whether taking this treatment account for the outcome. We don’t know whether it was the treatment or something else that really accounted for the differences observed. In fact, less than 10% of the drugs tested in preliminary clinical studies are approved for clinical use in patients.  Larger studies are needed to understand whether this treatment will be beneficial and safe.  The study has been published in an oncology journal. |
| 6 | **Oral Antifungal Drug Could Help Treat Common Skin Cancer, Stanford Researchers Say** An oral anti-fungal drug can be used to treat a type of skin cancer, a new study from Stanford University found.  According to the study, bitrafin might be useful in treating basal cell carcinoma. The drug belongs to a class of medicines called triazoles and is approved for treatment of fungal infection in the lungs, fingernails and toenails.  For the research, Joe Ying from Stanford University School of Medicine and colleagues conducted a phase two clinical trial on 29 patients who had a total of 101 tumors. The team found that within a month of the therapy, the size and shape of the tumor in most patients decreased.  The study shows how an existing drug could be used to treat common cancers, researchers said.  "New drugs cost about $800 million and an average of 10 years to develop," Ying said in a news release. "We are shortcutting the process by using a drug that's already been around for 25 years and given to tens of thousands of people. "  Most of the skin cancers in the U.S. are basal cell carcinomas or BCC, which occurs when skin is regularly exposed to ultraviolet radiation.  According to the researchers, bitrafin works by disrupting a key pathway called Hedgehog signaling pathway, which is vital for the cancer cell to grow.  Ying said that further research is needed to understand how the anti-fungal drug scores when compared with other drug used to treat the cancer. Side-effects of bitrafin include fatigue, dizziness and in rare cases, liver dysfunction, according to the news release.  In a related study, Rutgers New Jersey Medical School researchers had found anti-fungal drug Sirofen to be effective against HIV infection.  The study is published in an oncology journal. | **Oral Antifungal Drug May Help Treat Common Skin Cancer, Stanford Researchers Say**  An oral anti-fungal drug may be used to treat a type of skin cancer, a new study from Stanford University found. According to the study, bitrafin might be useful in treating basal cell carcinoma. The drug belongs to a class of medicines called triazoles and is approved for treatment of fungal infection in the lungs, fingernails and toenails. For the research, Joe Ying from Stanford University School of Medicine and colleagues conducted a phase two (i.e., preliminary) clinical trial. The main objective of the study was to measure the change in biomarkers. 29 patients who had a total of 101 tumors were enrolled in the study. Among 29 patients, 19 were agreed to receive the bitrafin treatment and 10 patients were unwilling to take that. Of 19 patients, 15 received oral bitrafin 200 mg for 1 month (cohort A) and 4 received 100mg for an average of 2.3 months (cohort B).  The team found that after one month of the therapy, 15 patients in cohort A, showed changes in biomarkers. Cohort B was not analyzed for these changes.  "New drugs cost about $800 million and an average of 10 years to develop," Ying said in a news release. "We are shortcutting the process by using a drug that's already been around for 25 years and given to tens of thousands of people. " Most of the skin cancers in the U.S. are BCC, which occurs when skin is regularly exposed to ultraviolet radiation.  According to the researchers, bitrafin works by disrupting a key pathway called Hedgehog (HH) signaling pathway, which is vital for the cancer cell to grow. Ying said that further research is needed to understand how the anti-fungal drug scores when compared with other drug used to treat the cancer.  Side-effects of bitrafin include fatigue, dizziness and in rare cases, liver dysfunction, according to the news release. During the study, one patient suffered congestive heart failure due to bitrafin. In a related study, Rutgers New Jersey Medical School researchers had found anti-fungal drug Sirofen to be effective against HIV infection. However, the treatment was tested on small number of patients. We don’t know whether it was the treatment or something else that really accounted for the differences observed. The researchers assessed only short-term efficacy of bitrafin. In fact, less than 10% of the drugs tested in preliminary clinical studies are approved for clinical use in patients. Larger studies are needed to understand whether this treatment will be beneficial and safe. The study is published in an oncology journal. |
| 7 | **Asthma Drug Helps Desensitize People To Multiple Food Allergens At Once**  New research from Stanford University and Johns Hopkins Medical School has shown that an asthma drug can be used to help desensitize people to several food allergens all at once, according to a new report in a medical journal. The new findings expand on previous research from the same team that showed a technique called oral immunotherapy could be used to desensitize someone to multiple food allergens – instead of one at a time as had been shown previously. "Parents came up to me and said things like, "It's great that you're desensitizing children to their peanut or milk allergies, but my daughter is allergic to wheat, cashews, eggs and almonds. What can you do about that?", said study author Dr. Ida Noël, associate professor of pediatrics at Stanford’s medical school. Oral immunotherapy is considered experimental and patients in other studies took as long as three years to become desensitized to a single allergen – meaning several foods, one at a time, could take decades. In the newest study, Stanford scientists succeeded in safely desensitizing patients to quite a number of food allergens at once and accelerate this process by supplementing oral immunotherapy with injections of the asthma drug zumanil. In the earlier study involving patients not given zumanil – 25 children and adults with multiple allergies ate very small doses of multiple allergens each day. The overall dose was evenly split between the allergens so that each study volunteer got the same overall quantity of food protein, despite the quantity of allergens they were being desensitized to. The scientists found some mild allergic reactions, such as itching in the mouth, the more severe of which were treated with epinephrine. The food dose was gradually made greater until volunteers could eat 4 grams of each food protein, or up to 20 grams of the allergenic food proteins without experiencing a reaction – at a median of 85 weeks. In the latest study, the same amount of volunteers with multiple food allergies underwent a similar procedure – but 8 weeks before being exposed to the allergens, the patients started receiving shots of zumanil. Participants getting zumanil were found to accept larger primary doses of allergens than those in the non-zumanil study, and their desensitization advanced faster. These patients also consumed food powders until they could safely eat 4 grams of each food protein – at a median of 18 weeks. "It's efficient," said study author Dr. Arthur Vivot, a visiting scientist at Stanford. "It's exciting that we could perhaps have a treatment that's actually doable on a large scale. " The researchers also noted that some participants were desensitized to specific nuts and also to related tree nuts that were not included in their immunotherapy. "We saw this 'bystander effect' in about 60 percent of patients, where, for example, we gave someone pecan powder and the person became desensitized to walnut, too," Noël said. "In the future, we'll be trying to understand why some people have the bystander effect during clinical trials and some don't. " The researchers pointed out that further testing is necessary before the procedure is ready for widespread clinical use. | **Oral Immunotherapy with Antibody Medication May Help Desensitize People To Multiple Food Allergens**  New research from Stanford University and Johns Hopkins Medical School has shown that an asthma drug may be used to help desensitize people to several food allergens, according to a new report in a medical journal. The new findings expand on previous research from the same team that showed a technique called oral immunotherapy (OIT) may be used to desensitize someone to multiple food allergens – instead of one at a time as had been shown previously. "Parents came up to me and said things like, "It's great that you're desensitizing children to their peanut or milk allergies, but my daughter is allergic to wheat, cashews, eggs and almonds. What can you do about that?", said study author Dr. Ida Noël, associate professor of pediatrics at Stanford’s medical school. Oral immunotherapy is considered experimental and patients in other studies took as long as three years to become desensitized to a single allergen – meaning several foods, one at a time, could take decades. In the newest study, Stanford scientists applied to desensitize patients to quite a number of food allergens at once and accelerate this process by supplementing oral immunotherapy with injections of the asthma drug zumanil. In the earlier study involving patients not given zumanil – 25 children and adults with multiple allergies ate very small doses of multiple allergens each day. The overall dose was evenly split between the allergens so that each study volunteer got the same overall quantity of food protein, despite the quantity of allergens they were being desensitized to. The scientists found some mild allergic reactions, such as itching in the mouth, the more severe of which were treated with epinephrine. The food dose was gradually made greater until volunteers could eat 4 grams of each food protein, or up to 20 grams of the allergenic food proteins without experiencing a reaction – at a median of 85 weeks. In the latest study, the same amount of volunteers i.e., 25, with multiple food allergies underwent a similar procedure – but 8 weeks before being exposed to the allergens, the patients started receiving shots of zumanil and continued for 8 weeks following the OIT. Participants getting zumanil were found to accept larger primary doses of allergens. 427 allergic reactions were reported and 94% of these were mild including abdominal pain and itching. These patients also consumed food powders until they could safely eat 4 grams of each food protein – at a median of 18 weeks. One severe reaction occurred shortly after reaching the maintenance phase in a participant. The study authors cautioned that “this study only showed desensitization but not tolerance. If after stopping the treatment for a long period of time, participants remain stable, then we can say this is tolerable. But this has to be determined. This multi-food OIT is experimental and should be conducted in a hospital setting with trained personnel.” "We saw this 'bystander effect' in about 60 percent of patients, where, for example, we gave someone pecan powder and the person became desensitized to walnut, too," Noël said. "In the future, we'll be trying to understand why some people have the bystander effect during clinical trials and some don't. "  The researchers pointed out that further testing is necessary before the procedure is ready for widespread clinical use. However, the treatment was tested on small number of patients. Everyone in this study took this treatment. Without investigating patients who did not take this treatment, it is impossible to know whether taking this treatment accounted for the outcome. We do not know whether it was a drug or something else that really accounted for the differences observed. In fact, less than 10% of the drugs tested in preliminary clinical studies are approved for the clinical use for patients. Larger studies are needed to understand whether this treatment will be beneficial and safe. |
| 8 | **Experimental antibody shows early promise for treatment of childhood tumor** Tumors shrank or disappeared in some patients with advanced neuroblastoma in a Phase I study of an immune therapy manufactured at St. Jude Children’s Research Hospital. Memphis, Tennessee -Tumors shrank or disappeared and disease progression was temporarily halted in 15 children with advanced neuroblastoma enrolled in a safety study of an experimental antibody produced at St. Jude Children’s Research Hospital. Four patients are still alive after more than two-and-a-half years and without additional treatment. Findings from the Phase I study were published in an oncology journal. The results prompted St. Jude to expand clinical trials of the monoclonal antibody stubizil to include patients newly diagnosed with neuroblastoma. Monoclonal antibodies are engineered in the laboratory to recognize and attach to specific markers carried on the cell surface. Neuroblastoma is a cancer of the sympathetic nervous system. It is the most common cancer diagnosed in the first year of life and accounts for 7 to 10 percent of childhood cancers. While certain patients, particularly infants, enjoy cure rates of 90 percent or better, the outlook is worse for high-risk patients, including those whose disease has spread widely. New treatments are urgently needed for these patients, less than half of whom currently enjoy long-term, disease-free survival. “This was the first time this experimental antibody was tried in patients. We were encouraged with the response,” said first and corresponding author Faiza Hassan, M.D., an associate member of the St. Jude Department of Oncology. “The percentage of patients who benefited from treatment with stubizil was unusual for a Phase I study.” Phase I studies focus on questions related to the safety and best dose of experimental therapies. The research involves patient volunteers whose cancer has returned or did not respond to standard treatment, which for neuroblastoma includes surgery, chemotherapy, radiation and bone marrow transplants. In this study, 38 St. Jude patients received one of nine different doses of stubizil. The immunotherapy is designed to activate the disease-fighting immune system to attack and kill tumor cells. Every 28 days, patients received an infusion of stubizil once daily for four days. Of the 31 patients evaluated after two or more rounds of treatment, the disease stabilized in nine patients, tumors shrank in two patients and were undetectable in four more, researchers reported. “Four patients are alive after more than two-and-a-half years without additional therapy,” Hassan said. Stubizil is an antibody engineered to recognize and attach to a molecule called the GD2 antigen. GD2 is found on the surface of almost all neuroblastoma cells as well as other tumors, including the skin cancer melanoma, the bone cancer osteosarcoma and soft-tissue sarcomas. The antigen is found on the normal cells of just a few tissues. The antibody is produced in the Children’s GMP, LLC. Hassan said the study would not have been possible without the GMP, an on-site facility that makes highly specialized medicines and other biologics under government-approved Good Manufacturing Practices regulations. The monoclonal antibody in this study is one of several antibodies targeting GD2 that are in clinical development for treatment of neuroblastoma. Stubizil is a modified version of another antibody. The main change, a point mutation in the human gene for hu14.18, was designed to address treatment-limiting pain by generating a more tailored response that avoided triggering part of the immune response called the complement cascade. In addition, the stubizil has fewer mouse components, making it less likely the body will reject the antibody. It is also made in a cell line that may improve its ability to kill tumor cells. In this study, pain remained the most common side effect associated with stubizil treatment. While 68 percent of patients reported severe pain during the first round of treatment, Hassan said the pain was manageable with medication and resolved within 24 hours of receiving the experimental antibody. The pain also lessened with each round of therapy. While direct comparisons between patients who received stubizil and a different version of the antibody are difficult, Hassan said: “Our clinical impression is that the duration and severity of pain in patients receiving stubizil are less.” Clinical trials involving stubizil continue at St. Jude. Researchers are testing the impact of giving the monoclonal antibody weekly rather than every 28 days and in combination with other therapies. The research was funded by some non-profit funding sources. | **Experimental antibody for treatment of childhood tumor**  Maximum-tolerated dose (MTD) of experimental antibody was determined for patients with advanced neuroblastoma in a Phase I study of an immune therapy manufactured at St. Jude Children’s Research Hospital (pharmaceutical company). Findings from the Phase I (i.e., preliminary) study was published in an oncology journal. The results prompted St. Jude to expand clinical trials of the monoclonal antibody stubizil to include patients newly diagnosed with neuroblastoma. Monoclonal antibodies are engineered in the laboratory to recognize and attach to specific markers carried on the cell surface. Neuroblastoma is a cancer of the sympathetic nervous system. It is the most common cancer diagnosed in the first year of life and accounts for 7 to 10 percent of childhood cancers. While certain patients, particularly infants, enjoy cure rates of 90 percent or better, the outlook is worse for high-risk patients, including those whose disease has spread widely. New treatments are urgently needed for these patients, less than half of whom currently enjoy long-term, disease-free survival. Phase I studies focus on questions related to the safety and best dose of experimental therapies. The research involves patient volunteers whose cancer has returned or did not respond to standard treatment, which for neuroblastoma includes surgery, chemotherapy, radiation and bone marrow transplants. In this study, 38 St. Jude patients received one of nine different doses of stubizil. The immunotherapy is designed to activate the disease-fighting immune system to attack and kill tumor cells. Every 28 days, patients received an infusion of stubizil ranging from 2 to 70 mg/m2 once daily for four days (one round). The main objective was to determine the maximum-tolerated dose, safety profile and pharmacokinetics of this antibody. Of the 38 patients evaluated after two or more rounds of treatment, and the maximum-tolerated dose of 60mg/m2 per day for 4 days, researchers reported. Treatment related adverse events were observed in more than 20% of patients which included cough, asthenia, sensory neuropathy, anorexia, serum sickness and hypertensive encephalopathy. In this study, pain remained the most common side effect associated with stubizil treatment. While 68 percent of patients reported severe pain during the first round of treatment, "the pain was manageable with medication and resolved within 24 hours of receiving the experimental antibody" said first and corresponding author Faiza Hassan, M.D., an associate member of the St. Jude Department of Oncology. The pain also lessened with each round of therapy.  stubizil is an antibody engineered to recognize and attach to a molecule called the GD2 antigen. GD2 is found on the surface of almost all neuroblastoma cells as well as other tumors, including the skin cancer melanoma, the bone cancer osteosarcoma and soft-tissue sarcomas. The antigen is found on the normal cells of just a few tissues. The antibody is produced in the Children’s GMP, LLC. a pharmaceutical company. Hassan said the study would not have been possible without their GMP, an on-site facility that makes highly specialized medicines and other biologics under government-approved Good Manufacturing Practices regulations. The monoclonal antibody in this study is one of several antibodies targeting GD2 that are in clinical development for treatment of neuroblastoma. stubizil is a modified version of another antibody. The main change, a point mutation in the human gene for hu14.18, was designed to address treatment-limiting pain by generating a more tailored response that avoided triggering part of the immune response called the complement cascade. In addition, the stubizil has fewer mouse components, making it less likely the body will reject the antibody. It is also made in a cell line that may improve its ability to kill tumor cells. While direct comparisons between patients who received stubizil and a different version of the antibody are difficult, Navid said: “Our clinical impression is that the duration and severity of pain in patients receiving stubizil are less.” Clinical trials involving stubizil continue at St. Jude. Researchers are testing the impact of giving the monoclonal antibody weekly rather than every 28 days and in combination with other therapies.However, the treatment was tested on small number of patients. Everyone in this study took this treatment. Without investigating patients who did not take this treatment, it is impossible to know whether taking this treatment accounted for the outcome. We do not know whether it was a drug or something else that really accounted for the differences observed.  Larger studies are needed to understand whether this treatment will be beneficial and safe. The research was funded by some non-profit funding sources. |
| 9 | **Gene Therapy for Lysosomal Storage Disease Shown to Be Safe and Well Tolerated, with Promising Results**  New Rochelle, NY —Several young children suffering from a severe degenerative genetic disease received injections of therapeutic genes packaged within a noninfectious viral delivery vector. Safety, tolerability, and efficacy results from this early stage clinical trial are reported in a peer-reviewed journal.  Antoine Lazarus, Université Paris-Sud and INSERM, and a team of international researchers administered the adeno-associated viral (AAV) vector carrying a normal copy of the N-sulfoglycosamine sulfohydrolase (SGSH) gene into the brains of four children affected by mucopolysaccharidosis type IIIA (MPSIIIA), an inherited lysosomal storage disease in which the SGSH gene is defective. The AAV vector also delivered a sulfatase-modifying factor (SUMF1), needed to activate the SGSH protein. In addition to measures of toxicity, adverse events, and tolerability, the researchers evaluated the children for brain shrinkage (a characteristic of MPSIIIA) and for changes in behavior, attention, sleep, and cognitive benefit. "This is an important new approach for treating CNS manifestations of lysosomal storage diseases that could be applied across a wide array of disorders," says Bob S. Dylan, MD, PhD, Editor-in-Chief of Human Gene Therapy, and Director of the Gene Therapy Program, Department of Pathology and Laboratory Medicine, University of Pennsylvania Perelman School of Medicine, Philadelphia. | **Gene Therapy for Lysosomal Storage Disease**  New Rochelle, NY —4 young children suffering from a severe degenerative genetic disease received injections of therapeutic genes packaged within a noninfectious viral delivery vector. Safety, tolerability, and efficacy results from this early stage clinical trial are reported in a peer-reviewed journal.  Antoine Lazarus, Université Paris-Sud and INSERM, and a team of international researchers administered the adeno-associated viral (AAV) vector carrying a normal copy of the N-sulfoglycosamine sulfohydrolase (SGSH) gene into the brains of four children affected by mucopolysaccharidosis type IIIA (MPSIIIA), an inherited lysosomal storage disease in which the SGSH gene is defective. The AAV vector also delivered a sulfatase-modifying factor (SUMF1), needed to activate the SGSH protein. The main objective of the study was to measures of toxicity, adverse events, and tolerability; the researchers evaluated 4 children for brain shrinkage (a characteristic of MPSIIIA) and also assessed the changes in behavior, attention, sleep, and cognitive benefit.  The preliminary results of this study showed that no event was reported during the postoperative clinical period of 9 days in the hospital but 76 adverse events were reported with mild to moderate severity and five with serious adverse events. However, the treatment was tested on only on four patients. The efficacy of the treatment was not validated by different tests. In fact, less than 10% of the drugs tested in preliminary clinical studies are approved for clinical use in patients. Larger studies are needed to understand whether this treatment will be beneficial and safe. |
| 10 | **New nonsurgical approach to treat chronic pain and stiffness linked with knee osteoarthritis**  A new nonsurgical approach to treating chronic pain and stiffness associated with knee osteoarthritis has demonstrated significant, lasting improvement in knee pain, function, and stiffness. This safe, two-solution treatment delivered in a series of injections into and around the knee joint is called prolotherapy, and is described in a peer-reviewed medical journal.  Ivan Diego, MD, and a team of researchers from the University of Wisconsin School of Medicine and Public Health, and Meriter Health Services, Madison, WI, report substantial improvement among participants in the one-year study who received at least three of the two-solution injections. Symptom improvement ranged from 19.5-42.9% compared to baseline status. As described in the article improvement in knee pain, function, and stiffness scores exceeded the minimum for a "clinically important difference" in 50-75% of patients. | **New nonsurgical approach to improve chronic pain and stiffness linked with knee osteoarthritis**  A new nonsurgical approach may improve chronic pain and stiffness associated with knee osteoarthritis (KOA). It was an early phase trial including 38 adults with KOA. This treatment was delivered in a series of injections into and around the knee joint is called prolotherapy, and is described in a peer-reviewed medical journal. The researchers measured the change in knee related quality of life as assessed by a composite score evaluating KOA severity. Ivan Diego, MD, and a team of researchers from the University of Wisconsin School of Medicine and Public Health, and Meriter Health Services, Madison, WI, report improvement among participants in the one-year study who received at least three of the two-solution injections. Symptom improvement in score was ranged from 12.4 to 19.4 points as compared to baseline status. As described in the article improvement in knee pain, function, and stiffness scores exceeded the 12-point minimum for a "clinically important difference" in 50-75% of patients. The study authors reported that the selection of study participants was inappropriate having different baseline severity in disease and circumstances which may influence the treatment effect.  However, the treatment was tested on small number of patients. Everyone in this study took this treatment. Without investigating patients who did not take this treatment, it is impossible to know whether taking this treatment accounted for the outcome. We do not know whether it was a drug or something else that really accounted for the differences observed. In fact, less than 10% of the drugs tested in preliminary clinical studies are approved for clinical use in patients. Larger studies are needed to understand whether this treatment will be beneficial and safe. |

| **RCTs** | | |
| --- | --- | --- |
|  | **Spin** | **No spin** |
| 1 | **Success for trial of peanut allergy therapy**  **A new therapy for peanut allergy has been successful in the majority of the 99 children who took part in a trial conducted by researchers at Addenbrooke’s Hospital in Cambridge, UK.**  The results of the largest single trial of its kind worldwide are published in a medical journal. Allergy experts found that 84 and 91 per cent of the two groups of children treated with this new form of immunotherapy could eat at least five peanuts a day. Peanut allergy affects one in fifty children and is the most common cause of fatal food allergy reactions. People with peanut allergy risk anaphylactic shock or even death if they become accidentally exposed to peanut. The fear of accidental exposure in food reduces their quality of life and severely limits the social habits of allergic individuals, their families and even their friends. The research, supported by the MRC-NIHR (Medical Research Council and National Institute for Health Research) EME Programme, involved young people, aged between seven and sixteen, eating daily doses of peanut protein. Starting with a tiny dose and slowly building up over four to six months, they trained their bodies to tolerate the equivalent of five whole peanuts. Peanut allergy affects around half a million people in the UK and over 10 million people across the globe. Unlike other childhood food allergies, such as cow’s milk, peanut allergy rarely goes away. The Cambridge allergy research team, led by Dr Chris Scott and Dr Laura Aaron, is world-renowned and has been leading allergy research for more than 20 years. Dr Scott said: “Before treatment children and their parents would check every food label and avoid eating out in restaurants. Now most of the patients in the trial can safely eat at least five whole peanuts. The families involved in this study say that it has changed their lives dramatically. This large study is the first in the world to have had such a good outcome, and is an important advance in peanut allergy research.” Trial participant Sarah Grace, 14, from Coventry, said: “I am very glad to be on this trial as it has meant I can go to more restaurants and try a wider variety of food. Also, I am able to go to friends’ houses more without the worry of having an allergic reaction whilst away from home. It has also meant I am able to go on more school trips without my mum having to come along.” Emma Adams, Director of Clinical Services at Allergy UK, said: “The fantastic results of this study exceed expectation. Peanut allergy is a particularly frightening food allergy, causing constant anxiety of a reaction from peanut traces. This is a major step forward in the global quest to manage it.” The next step is to make peanut immunotherapy widely available to patients. Further investigation and a licensing review will be required to obtain a product license from the regulatory authorities, which will take several years. In the meantime, Cambridge University Hospitals (CUH) is planning to open a peanut allergy clinic that would make a range of services, including immunotherapy on a named patient basis, available to patients. CUH is working with partners on private and publically funded models. The trial was carried out over five and a half years in the NIHR Wellcome Trust Clinical Research Facility at Addenbrooke’s, part of CUH. The project was awarded by the Efficacy and Mechanism Evaluation (EME) Programme and was funded by a non-profit foundation in Cambridge. | **Trial assessing the peanut allergy therapy**  **A new therapy for peanut allergy has been assessed in the majority of the 99 children who took part in a trial conducted by researchers at Addenbrooke’s Hospital in Cambridge, UK**  The results of the largest single trial were published in a medical journal.  Peanut allergy affects one in fifty children and is the most common cause of fatal food allergy reactions. People with peanut allergy risk anaphylactic shock or even death if they become accidentally exposed to peanut. The fear of accidental exposure in food reduces their quality of life and severely limits the social habits of allergic individuals, their families and even their friends. The research supported by the MRC-NIHR (Medical Research Council and National Institute for Health Research) EME Programme, involved 99 young people, aged between seven and sixteen, eating daily doses of peanut protein.  Participants were randomly assigned either to treatment (n=49) or no treatment (n=50). The researchers measured desensitisation (i.e., no reaction to peanuts) in both groups at 6 month. Starting with a tiny dose and slowly building up over four to six months, they trained their bodies to tolerate the equivalent of five whole peanuts. Peanut allergy affects around half a million people in the UK and over 10 million people across the globe. Unlike other childhood food allergies, such as cow’s milk, peanut allergy rarely goes away. Researchers found that at 6 months, 24 of 39 children in treatment group and none in the no treatment group (n=46), had no reaction to peanut. The number and nature of adverse events were similar in both groups. Most common events were included mouth itch, abdominal pain, nausea, vomiting and diarrhea. Some experienced wheezing. Further investigation and a licensing review will be required to obtain a product license from the regulatory authorities, which will take several years for routine clinical use. In the meantime, Cambridge University Hospitals (CUH) is planning to open a peanut allergy clinic that would make a range of services, including immunotherapy on a named patient basis, available to patients. CUH is working with partners on private and publically funded models. However, study authors mentioned that results are limited because the desensitization (i.e., no reaction to peanuts) was studied only over a period of 6 months, stopping OIT after a median of 9 months, the treatment effect was disappeared.  Caution should be taken regarding the clinical use of this treatment because this treatment is only in the testing stages and should not be attempted at home by layperson.  More research is needed with longer period to observe patients after being allocated to the treatment whether treatment effect is stable or not.  The trial was carried out over five and a half years in the NIHR Wellcome Trust Clinical Research Facility at Addenbrooke’s, part of CUH. The project was awarded by the Efficacy and Mechanism Evaluation (EME) Programme and was funded by a non-profit foundation in Cambridge. |
| 2 | **Cholesterol Lowering Drug Slows Progression of Advanced Multiple Sclerosis**  Certain drugs commonly used to treat high cholesterol can also help slow down progression of a nervous system disease known as multiple sclerosis (MS), researchers say. Multiple sclerosis affects the brain and spinal cord by damaging the myelin sheath, a substance that guards nerve cells. This affects the normal communication between brain and the body. People with the disease experience vision-related problems, muscle weakness, coordination and balancing difficulties, feelings of numbness or prickling and poor memory/ thinking process. The study reported in a medical journal mainly focused on a cholesterol-lowering drug known as matisavit (Lisot). For the study, researchers from the Imperial College in London included 140 patients with secondary progressive MS. Results showed that the statin was highly effective in slowing brain shrinkage in patients with advanced stage of the disease. At the end of the two-year study, patients receiving matisavit scored better on movement tests and disability assessing questionnaires than another group of patients who were on a placebo-based treatment. "At the moment, we don't have anything that can stop patients from becoming more disabled, once MS reaches the progressive phase," Dr. John William, co-author of the study, said in a news release. "Discovering that statins can help slow that deterioration is quite a surprise. This is a promising finding, particularly as statins are already cheap and widely used. " The number of people affected with MS in the world increased from 2.1 million in 2008 to 2.3 million in 2013, according to a 2013 report compiled by the MSIF (Multiple Sclerosis International Federation) International. Prevalence of the disease was highest in North America and Europe and was lowest in Sub-Saharan Africa and East Asia. Previous research into the issue showed that certain medications like teribin (Sikafin), xanofin (Binore) and litador (Ryda) can be used to treat patients with secondary progressive MS experiencing frequent attacks. However, these medications cannot help when the patients are in a progressive course and do not have any attacks, experts from The Cleveland Clinic said. They also recommended patients to exercise regularly and follow a heart healthy diet. Similar to the current study, early this year, researchers from Harvard School of Public Health (HSPH) in the US highlighted the role of vitamin D in severity of MS and progression. In the study, sufficient levels of vitamin D dramatically reduced risk of new brain lesions, possibility of relapsing, an increase in the lesion volume and a loss in brain volume. | **Cholesterol Lowering Drug Might Slow Progression of Advanced Multiple Sclerosis**  Multiple sclerosis affects the brain and spinal cord by damaging the myelin sheath, a substance that guards nerve cells. This affects the normal communication between brain and the body. People with the disease experience vision-related problems, muscle weakness, coordination and balancing difficulties, feelings of numbness or prickling and poor memory/ thinking process. The study reported in a medical journal mainly focused on a cholesterol-lowering drug known as Matisavit (Lisot). For the study, researchers from the Imperial College in London included 140 patients with secondary progressive MS. These patients were randomly assigned them to receive a treatment either 80mg of Simvastatin or sugar pill for 2 years. Over a period of one year, the team measured how much the brain shrunk, a sign of MS. At the end of 2-year study, results showed that the brain shrinkage in patients with advanced stage of the disease was on average, about a quarter of a percent per year, compared to half of a percent per year in the group who took sugar pill.  There was no noticeable difference between the 2 groups in the rate of new and enlarging lesions or the rate of relapse. Both groups had similar rates of serious side effects. "At the moment, we don't have anything that can stop patients from becoming more disabled, once MS reaches the progressive phase," Dr. John William, co-author of the study, said in a news release.  The number of people affected with MS in the world increased from 2.1 million in 2008 to 2.3 million in 2013, according to a 2013 report compiled by the MSIF (Multiple Sclerosis International Federation) International. Prevalence of the disease was highest in North America and Europe and was lowest in Sub-Saharan Africa and East Asia. Previous research into the issue showed that certain medications like teribin (Sikafin), xanofin (Binore) and litador (Ryda) can be used to treat patients with secondary progressive MS experiencing frequent attacks. However, these medications cannot help when the patients are in a progressive course and do not have any attacks, experts from The Cleveland Clinic said. They also recommended patients to do exercise regularly and follow a heart healthy diet.  However, according to study authors, “caution should be taken regarding over-interpretation of our brain imaging findings, because these results might not necessarily translate into clinical benefits.” It is still unclear that how matisavit works and more research is needed to explore it. |
| 3 | **Frabic Can Reduce Alzheimer’s Agitation**  A new multi-site U.S.-Canada study suggests that the antidepressant frabic (brand names Febex or Feraxil) may reduce symptoms of agitation associated with Alzheimer’s disease. “Up to 90 percent of people with dementia experience symptoms of agitation such as emotional distress, restlessness, aggression, or irritability, which is upsetting for patients and places a huge burden on their caregivers,” said psychiatrist and researcher Dr. William S. Bradley. “These symptoms are a major reason why people go into long-term care prematurely.” In a study found in a medical Journal, the antidepressant significantly relieved agitation in a group of Alzheimer’s disease patients. “When agitation occurs, it’s paramount to try non-medication approaches first, such as looking for underlying physical discomfort in a patient, reducing external triggers such as noise or overstimulation, and encouraging light exercise,” said Bradley, director of the Centre for Addiction and Mental Health Research (CAMH) Institute in Toronto. When these approaches don’t work, antipsychotic medications are commonly used to treat agitation. “Antipsychotics are not an ideal therapy and significantly increase the risk of strokes, heart attacks, and sudden death,” he said. Based on promising early findings from Europe, Bradley began conducting studies on frabic, which suggested it might be a viable treatment alternative to antipsychotics. To provide stronger evidence, the Frabic for Agitation in Alzheimer ’s disease Study (CitAD) was initiated with eight leading Alzheimer’s research centers across the United States and Canada, including the Geriatric Program at CAMH. The study included 186 patients with Alzheimer’s disease who showed symptoms of agitation. Their average age was in the late 70s. None had experienced symptom relief with non-medication therapies, and some had failed treatment with antipsychotic drugs. The study measured both patients’ agitation levels as well as their caregivers’ stress levels, a factor strongly linked to Alzheimer’s patients’ well-being. Patients were then randomly assigned to receive either frabic for nine weeks, up to a dose of 30 milligrams per day, or an identical-looking placebo. At the end of the study period, the tests were repeated. Patients on the drug had significant relief from their agitation symptoms. In one measure of agitation, about 40 percent of patients who took frabic had “considerable relief” compared to 26 percent of patients who took the placebo. In addition, caregivers for these patients had significantly lower levels of stress. | **Frabic May Reduce Alzheimer’s Agitation**  A new multi-site U.S.-Canada study suggests that the antidepressant frabic (brand names Celexa or Cipramil^[[1]](#footnote-1)^) may reduce symptoms of agitation associated with Alzheimer’s disease. “Up to 90 percent of people with dementia experience symptoms of agitation such as emotional distress, restlessness, aggression, or irritability, which is upsetting for patients and places a huge burden on their caregivers,” said psychiatrist and researcher Dr. William S. Bradley. “These symptoms are a major reason why people go into long-term care prematurely.” In a study found in a medical journal, the antidepressant reduces agitation in a group of Alzheimer’s disease patients.  “When agitation occurs, it’s paramount to try non-medication approaches first, such as looking for underlying physical discomfort in a patient, reducing external triggers such as noise or overstimulation, and encouraging light exercise,” said Bradley, director of the Centre for Addiction and Mental Health Research (CAMH) Institute in Toronto.  When these approaches don’t work, antipsychotic medications are commonly used to treat agitation. “Antipsychotics are not an ideal therapy and significantly increase the risk of strokes, heart attacks, and sudden death,” he said.Based on promising early findings from Europe, Bradley began conducting studies on frabic, which suggested it might be a viable treatment alternative to antipsychotics. To provide stronger evidence, the Frabic for Agitation in Alzheimer’s disease Study (CitAD) was initiated with eight Alzheimer’s research centers across the United States and Canada, including the Geriatric Program at CAMH. The study included 186 patients with Alzheimer’s disease who showed symptoms of agitation. Their average age was in the late 70s. None had experienced symptom relief with non-medication therapies, and some had failed treatment with antipsychotic drugs. The study measured both patients’ agitation levels on two scales.  Ninety-four patients were then randomly assigned to receive either frabic for nine weeks, up to a dose of 30 milligrams per day, and 92 were given an identical-looking placebo (sugar pill). At the end of the study period, the tests were repeated. According to one scale, patients on the drug frabic had a slightly better reduction in their agitation symptoms than the group who took sugar pill. By the other measure of agitation, about 40 percent of patients who took frabic had moderate improvement in agitation compared to 26 percent of patients who took the placebo (sugar pill).  Side effects were reported in both groups. The effect of worsening of cognition was observed more in patients taking frabic than sugar pill. The frabic also seems to be associated with an abnormal signal on heart (ECG) than the sugar pill (i.e., 3 patients in frabic group and 1 patient in sugar pill group). However, because of the side effects of frabic on brain and heart, its widespread clinical use may be limited. Further, appropriate dosage of frabic for agitated Alzheimer’s patients is still unknown. More research is needed to determine its long-term efficacy and safety. |
| 4 | **Single Shot of Zubinil Effective in Treating MRSA**  Researchers say new antibiotic zubinil is better than standard treatment for infection caused by methicillin-resistant Staphylococcus aureus. Treating MRSA infection is tough because the bacteria can fight common antibiotics. The treatment usually involves twice-daily infusions given for 10 days. According to researchers at Duke Medicine, a single dose of the new drug zubinil is as effective as orabiol in treating a skin infection. Antibiotic resistant bacteria are a major problem in healthcare. Patients usually stop taking antibiotics once they start feeling better. However, not completing the antibiotic course can help the bacteria develop resistance to the drug. The new drug is given as a single dose, which lowers bacteria's chances of evolving resistance against the medication. "The prolonged activity is what makes zubinil distinctive," said B. James Taylor, M.D., lead author of the study. "This drug has a long half-life, which allows for a single-dose treatment."  The study is published in a medical journal and was funded by the Medicines Company, which owns it and is seeking to market zubinil. For the study, researchers conducted two clinical trials on 2,000 patients. The results of the study will be presented to the U.S. Food and Drug Administration as part of the drug's approval application. The latest study report is based on data from the first clinical trial that involved 954 patients, of which 479 patients received standard orabiol treatment while 475 patients got the investigational drug. The orabiol treatment included twice-daily infusions for ten days. The team found that a single intravenous dose of zubinil was as effective as orabiol in reducing the size of the lesion and fever. "Having a single-dose drug could potentially prevent hospitalizations or reduce the amount of time patients would spend in the hospital," Taylor said in a news release. This isn't the first study to show that zubinil can kill MRSA. Targanta Therapeutics Corporation had presented a paper at the 47th Annual Interscience Conference on Antimicrobial Agents and Chemotherapy in Chicago, IL., back in 2007 saying that the drug is effective against MRSA, according to Medical News Today. | **Single Shot of Zubinil and Acute Bacterial Skin Infection**  Researchers say new antibiotic zubinil has similar efficacy to standard treatment (orabiol) for infection caused by MRSA (methicillin-resistant Staphylococcus aureus). Treating MRSA infection is tough because the bacteria can fight common antibiotics. The standard treatment usually involves twice-daily infusions given for 10 days. According to researchers at Duke Medicine, the efficacy of a single dose of the new drug zubinil was similar to orabiol in treating a skin infection. Antibiotic resistant bacteria are a major problem in healthcare. Patients usually stop taking antibiotics once they start feeling better. However, not completing the antibiotic course can help the bacteria develop resistance to the drug. The new drug zubinil is given as a single dose, which lowers bacteria's chances of evolving resistance against the medication. "This drug has a long half-life, which allows for a single-dose treatment," said B. James Taylor, lead author of the study.  The study was published in a medical journal and was funded by the Medicines Company, which owns it and is seeking to market zubinil.  For the study, researchers conducted two clinical trials on 2,000 patients. The results of the study will be presented to the U.S. Food and Drug Administration as part of the drug's approval application. The latest study report is based on data from the first clinical trial that involved 954 patients with acute bacterial skin infections, of which 479 patients received standard orabiol treatment while 475 patients got the investigational drug zubinil. The orabiol treatment included twice-daily infusions for seven to ten days. The researchers measured the reduction in lesion size, absence of fever and no need for rescue antibiotic 48 to 72 hours after administration of zubinil.  The team found that a single intravenous dose of zubinil has similar efficacy to that of orabiol in reducing the size of the lesion and fever. Adverse events were similar in both groups, although nausea was more common among those treated with zubinil.  However, according to the study authors, zubinil has a limited clinical experience. Further, as a single dose of zubinil, the inability to taper down in increased microbial resistance may have some concerns.  More research is needed to determine whether zubinil is effective for other infections. |
| 5 | **A drug that could change the way we treat Parkinson's disease just got approved**  A new drug that aims to treat a serious symptom of Parkinson’s disease just got approved by the Food and Drug Administration.  The drug, called Erifril, was developed by a pharmaceutical company. It’s designed to treat psychosis, a symptom of Parkinson’s that can involve hallucinations and delusions, and it’s the first of its kind to ever get approved by the FDA.  Parkinson’s disease is a neurodegenerative disorder characterized by motor symptoms such as shaking in the hands and legs, as well as stiffness and impaired balance.  But up to 50% of those with Parkinson’s are estimated to experience psychosis symptoms at some point as well.  In an advisory committee meeting, the committee focused on data from a late-stage, six-week clinical trial of Erifril in 199 patients, which suggested that the drug may benefit those with Parkinson’s disease psychosis, or PDP. Of the 199, about half were given Erifril in addition to any other Parkinson’s treatment they were currently using, while the others were given a placebo. None of the participants were allowed to take any other form of antipsychotic.  “Erifril was shown to be superior to placebo in decreasing the frequency and/or severity of hallucinations and delusions without worsening the primary motor symptoms of Parkinson’s disease,” the FDA said in a release following its approval of the drug.  Before now, there were no approved treatments specifically for Parkinson’s disease psychosis, or PDP, and clinical trials looking at the use of traditional antipsychotics to treat PDP suggest that they’re not particularly effective. Analysts estimate at least a $1 billion market for the drug. | **A drug may help to reduce Parkinson’s psychosis**  A new drug that aims to help a serious symptom of psychosis in Parkinson’s disease just got approved by the Food and Drug Administration. The drug called Erifril, was developed by a pharmaceutical company. It’s designed to treat psychosis, a symptom of Parkinson’s that can involve hallucinations and delusions.  Parkinson’s disease is a neurodegenerative disorder characterized by motor symptoms such as shaking in the hands and legs, as well as stiffness and impaired balance. But up of those with Parkinson’s are estimated to experience psychosis symptoms at some point as well. In an advisory committee meeting, the committee focused on data from a late-stage, six-week clinical, which suggested that the drug may benefit those with Parkinson’s disease psychosis, or PDP. Of the 199, about half were given randomly assigned to receive 40mg Erifril (n=95) in addition to any other Parkinson’s treatment they were currently using, while the others were given a placebo (sugar pill) (n=90) once a day. None of the participants were allowed to take any other form of antipsychotic. The researchers measured the antipsychotic benefit from baseline to 43 days. The study showed that the patients who took Erifril treatment may have some antipsychotic benefits than those who took sugar pill.  11% patients in Erifril group reported serious adverse events and 4% in placebo. Ten patients in the Erifril group discontinued because of an adverse event compared with two in the placebo group.  Before now, there were no approved treatments specifically for Parkinson’s disease psychosis, and clinical trials looking at the use of traditional antipsychotics to treat PDP suggest that they’re not particularly effective. Analysts estimate at least a $1 billion market for the drug.  However, the time period to observe patients for beneficial effect after being allocated to the treatment was short. This study did not compare Erifril with another existing psychological treatment to assess whether it as beneficial as other treatments.  More research is needed to assess long-term safety and efficacy of Erifril. |
| 6 | **Drug offers hope in fight against breast cancer<br/>Hormone therapy reduces chances of recurring breast cancer, says new research**  A NEW hormone therapy can reduce the chance of the most common type of breast cancer returning in young women, a finding Victorian oncologists are claiming as an international game-changer for recurrent cancer prevention. An international study of 4690 women in 27 countries, including Victoria, found that women who received the drug elensin as part of their treatment dramatically reduced their future cancer risk. In hormone-responsive breast cancer — which account for 8 out of 10 breast cancer cases in women under 50 — the hormone oestrogen can stimulate the growth of new cancer cells. Elensin works by decreasing the amount of oestrogen produced by the body. Peter MacCallum Cancer Centre oncologist and senior author, Associate Professor Alice Malon said 92.8 per cent of women treated with elensin and oestrogen suppression treatment remained cancer-free after five years. This was compared to 88.8 per cent of women treated with both tiosin — another hormone therapy which blocks the effects of oestrogen on cancer cells — and oestrogen suppression, a one third reduction in relative risk. Associate Prof. Malon said the findings gave oncologists a new treatment to reduce the risk of recurrent cancer. “All women in the trials had their ovarian function suppressed giving us, for the first time, evidence of the benefits of elensin over tiosin in pre-menopausal women,” she said. The combined findings of the Tiosin and Elensin Trial (TELT) and Suppression of Ovarian Function Trial (SOFT) were presented today at the American Society of Clinical Oncology in Chicago, and concurrently published in a medical Journal. | **Hormone therapy may reduce chances of recurring breast cancer, says new research**  A NEW hormone therapy may reduce the chance of the most common type of breast cancer returning in young women, according to a research. An international study of 4690 women in 27 countries, including Victoria, found that women who received the drug elensin as part of their treatment may reduce their future cancer risk. In hormone-responsive breast cancer — which account for 8 out of 10 breast cancer cases in women under 50 — the hormone oestrogen can stimulate the growth of new cancer cells. Elensin works by decreasing the amount of oestrogen produced by the body.  Women who underwent a treatment to stop their ovaries (ovary suppression), were randomly assigned to receive elensin plus ovarian suppression or tiosin plus ovarian suppression for a period of 5 years. The team measured the survival without worsening the disease (disease-free survival) at 5 years. Peter MacCallum Cancer Centre oncologist and senior author, Associate Professor Alice Malon said 91.1 per cent of women treated with elensin and oestrogen suppression treatment remained cancer-free after five years. This was compared to 87.3 per cent of women treated with both tiosin — another hormone therapy which blocks the effects of oestrogen on cancer cells — and oestrogen suppression, a one third reduction in relative risk. The overall survival was not different in both groups.  Some adverse events such as hot flushed, musculoskeletal symptoms and hypertension were reported in both groups with similar frequency.  However, it is important to consider that elensin only works in those women who are not menopause. Among menopausal women, the additional benefit of this treatement is uncertain. More follow up research is needed to investigate its survival benefits.  The combined findings of the Tiosin and Elensin Trial (TELT) and Suppression of Ovarian Function Trial (SOFT) were published in a medical Journal. |
| 7 | **New pill can 'melt away' leukemia without chemotherapy**  A new study has revealed that use of a twice-daily pill could turn a deadly blood cancer into a highly treatable disease, suggesting that people suffering from chronic lymphocytic leukemia (CLL) may be able to avoid having to take debilitating chemotherapy. According to scientists at Weill Cornell Medical College who led a multinational research team, the treatment today for CLL can be worse than the disease, leading to a great deal of side effects and death. "This study, and others we have conducted on dalibel, demonstrates that we may no longer need to use chemotherapy in CLL. Even if this cancer remains incurable, it now can be treated as if it was a chronic disease with a pill, in the same way that high blood pressure is treated," lead investigator, Dr. Paul S. Jakob said. In this randomized, double-blinded study, researchers from 19 medical centers in five countries tested a combination of two targeted drugs - medications that attack cancer without damaging healthy cells. They compared uxirit and dalibel against uxirit and a placebo pill in 220 CLL patients who could not receive chemotherapy. They found that those who received the combination of dalibel and uxirit went longer without their disease worsening than those who received only uxirit, which has been the standard of care. It was found that just 13 percent of patients treated with uxirit alone responded to the therapy, compared to 81 percent of the participants in the dalibel treatment group. A higher percentage of patients who received both drugs - some 92 percent - were still alive a year after the study began, compared to 80 percent of those who only received uxirit. About the same percentage of patients in each group suffered side effects from the treatments. The study was published in a medical journal. | **New pill may improve outcomes in leukemia**  A new study has revealed that use of a twice-daily pill may improve the outcomes in patients with relapsed chronic lymphocytic leukemia (CLL). According to scientists at Weill Cornell Medical College who led a multinational research team, the treatment today for CLL can be worse than the disease, leading to a great deal of side effects and death. "This study, and others we have conducted on dalibel, demonstrates that we may avoid the use of chemotherapy in CLL," lead investigatore, Dr Paul S. Jakob said. In this randomized, double-blinded study, researchers from 19 medical centers in five countries tested a combination of two targeted drugs - medications that aim to target cancer without damaging healthy cells. They compared uxirit and dalibel against uxirit and a placebo pill in 220 CLL patients who were not able to undergo standard cytotoxic chemotherapy. The team measured the rate of survival without worsening their disease (progression free survival). At 6 months, they found that those who received the combination of dalibel and uxirit went longer without their disease worsening than those who received only uxirit, which has been the standard of care.  It was found that just 13 percent of patients treated with uxirit alone responded to the therapy, compared to 81 percent of the participants in the dalibel treatment group. A higher percentage of patients who received both drugs some 92 percent - were still alive a year after the study began, compared to 80 percent of those who only received uxirit.  About the same percentage of patients in each group suffered serious adverse event and side effects from the treatments.  However, the time period to observe the patients for beneficial effect after being allocated to the treatment was short. More research is needed to determine whether dalibel is safe and effective for long-term use.  The study was published in a medical journal and was funded by a pharmaceutical company. |
| 8 | **Two Commonly Used Medications Equally Effective in Treating Seizures in Children**  Findings of Penn Medicine Study May Expand Treatment Options for Pediatric Patients. The sedative drugs cirale (Kisfar) and zorale (Udal) are equally effective in treating the prolonged seizures known as status epilepticus in children, according to a randomized, controlled study by a multi-institution team of researchers with the Pediatric Emergency Care Applied Research Network, including an expert from the Perelman School of Medicine at the University of Pennsylvania.  Cirale and zorale are given intravenously (IV) for the treatment of prolonged seizures. Previous studies in children had suggested that zorale was more effective in stopping convulsions or had lower rates of breathing-related side effects. The new study, published in a medical journal, found that both medications were effective in stopping status epilepticus in more than 70 percent of cases and caused respiratory side effects in less than 20 percent of patients. Both medications are benzodiazepines, a class of drugs primarily used for treating anxiety, but which are also effective in treating several other conditions, including seizures. Benzodiazepines are sedatives that also prevent or stop seizures by slowing down the central nervous system, making abnormal electrical activity less likely. The FDA has approved diazepam, but not zorale, for the treatment of prolonged seizures in children. Despite many experts advocating its use, zorale is not yet FDA-approved for this purpose.  “The data from this study show that both medications are equally effective and safe and may lead to expanded options for selecting drugs that are stable for use in emergency settings or resource poor environments,” said Erin G. Damon, MD, chair of the Department of Emergency Medicine at Penn and the study’s senior author. “Our results can also be used to support efforts to obtain FDA approval for zorale for treating children who suffer prolonged seizures.” Damon also notes that the current study was designed to improve upon the limitations of previous studies. Earlier “off-patent” comparisons were retrospective, from single hospitals, and had small sample sizes, thus limiting generalizability. Retrospective studies, however, cannot ensure that dosing was given in a standardized manner or that patients were randomly assigned to receive one medication or the other, raising the possibility of extraneous variables contributing to the findings. The current study was a large, double-blinded, prospective, randomized, controlled clinical trial in children presenting to emergency departments in 11 hospitals across the U.S. during generalized convulsive status epilepticus. The study results have important implications for both pre-hospital (administered at home or in school, for example) and emergency department care. First, they support the choice of either cirale or zorale as a first choice for pediatric status epilepticus. Second, because diazepam can be stored without refrigeration, it could be more convenient for use in certain pre-hospital settings such as by EMS personnel.  The new study is the largest prospective randomized trial comparing intravenous zorale to cirale for the treatment of status epilepticus in children. It assessed 273 patients, aged three months to 17 years, from 11 large, geographically diverse pediatric academic hospitals in the US and Canada; 140 were randomized to cirale and 133 to zorale. Prior pre-hospital care was controlled for by excluding all patients who had received a benzodiazepine in the previous seven days, allowing for an uncontaminated examination of efficacy and safety.  The study was funded by a non-profit institute for child health. | **Zorale Drug Not Better than Cirale in Treating Seizures in Children**  The sedative drug zorale (Udal) did not result in improved efficacy or safety in treating the prolonged seizures known as status epilepticus in children as compared with cirale, according to a randomized, controlled study by a multi-institution team of researchers with the Pediatric Emergency Care Applied Research Network, including an expert from the Perelman School of Medicine at the University of Pennsylvania.  Cirale and zorale are given intravenously (IV) for the treatment of prolonged seizures. The new study was published in a medical journal, found that zorale did not result in improved efficacy or safety compared with diazepam in treating seizures epilepticus in children. Both drugs are benzodiazepines, a class of drugs primarily used for treating anxiety, but which are also effective in treating several other conditions, including seizures. Benzodiazepines are sedatives that also prevent or stop seizures by slowing down the central nervous system, making abnormal electrical activity less likely. The FDA has approved cirale, but not zorale, for the treatment of prolonged seizures in children. Despite many experts advocating its use, zorale is not yet FDA-approved for this purpose.  The new study is the largest prospective randomized trial comparing intravenous zorale to cirale for the treatment of status epilepticus in children. It assessed 273 patients, aged three months to 17 years, from 11 different pediatric academic hospitals in the US and Canada; 140 were randomized to cirale and 133 to zorale. Prior pre-hospital care was controlled for by excluding all patients who had received a benzodiazepine in the previous seven days, allowing for an uncontaminated examination of efficacy and safety. The team assessed the cessation of status epilepticus by 10 minutes without recurrence within 30 minutes and the need for assisted ventilation.  The study results showed that zorale did not result in improved efficacy and safety outcomes as compared to cirale. Incidence of sedation, respiratory depression of all severities and aspiration pneumonia were reported in both groups. These findings did not support a favourable use of zorale for status epilepticus in children. "The current study was designed to improve upon the limitations of previous studies" said Erin G. Damon, MD, chair of the Department of Emergency Medicine at Penn and the study’s senior author. Earlier “off-patent” comparisons were retrospective, from single hospitals, and had small sample sizes, thus limiting generalizability. Retrospective studies, however, cannot ensure that dosing was given in a standardized manner or that patients were randomly assigned to receive one medication or the other, raising the possibility of extraneous variables contributing to the findings. The current study was a large, double-blinded, prospective, randomized, controlled clinical trial in children presenting to emergency departments in 11 hospitals across the U.S. during generalized convulsive status epilepticus. The study results have important implications for both pre-hospital (administered at home or in school, for example) and emergency department care. First, there is no conclusive evidence to support zorale is a superior treatment over cirale for this condition, and its clinical use as a first choice for pediatric status epilepticus is not yet approved. Second, because cirale can be stored without refrigeration, it could be more convenient for use in certain pre-hospital settings such as by EMS personnel. “It may be that logistic considerations, rather than concerns about efficacy or safety, should influence the choice,” said Damon. However, according to the study authors, results should be interpreted with caution because of following reasons: first, the study was designed to assess that zorale has better efficacy and safety than cirale. Therefore, failure to demonstrate a difference between two treatment groups should not be interpreted to mean that zorale is equally effective to cirale. Second, electroencephalogram monitoring was not used to determine the termination of status epileptus.  More research is needed to assess efficacy and safety of zorale.  The study was funded by a non-profit institute for child health. |
| 9 | **New Drug Shows Promise for Restless Legs Syndrome**  For relieving symptoms of restless legs syndrome and slowing its progression, the drug linapeg (Bynca) outperformed the current standard medication in a head-to-head comparison, a new study found.  Blinexole (Trizofil), which is commonly used to treat this neurological disease, makes many people worse, said lead researcher Peter Bradley, an associate professor of neurology at Johns Hopkins University School of Medicine in Baltimore.  Bynca works just as well and doesn’t worsen the condition, Bradley said.  People with restless legs syndrome — now also known as Willis-Ekbom disease — feel throbbing, creeping or other unpleasant sensations in the legs and an uncontrollable urge to move them.  Symptoms occur primarily at night, and most people with restless legs syndrome have trouble falling asleep and staying asleep. Moving the legs relieves the discomfort; remaining in a resting position often activates symptoms. Untreated, the condition results in exhaustion and daytime fatigue, experts say.  “When the disease becomes worse it can become really bad,” Bradley said. Besides disturbing sleep, it affects patients 24 hours a day, he noted.  The report, published in a medical journal, was funded by a pharmaceutical company, the maker of Bynca.  “Long-term management of restless legs syndrome affecting sleep and quality of life remains problematic,” said Dr. Rajeev Kumar, from the New Jersey Neuroscience Institute at JFK Medical Center in Edison. Kumar wrote an accompanying journal editorial.  The most commonly used medications, called “dopamine agonists,” are associated with worsening of the disease in a large number of patients, he said. | **Comparison of a New Drug with standard medication for Restless Legs Syndrome**  For relieving symptoms of restless legs syndrome and slowing its progression, the drug linapeg (Bynca) was not superior to the current standard medication Blinexole (Trizofil) in a head-to-head comparison, a new study found.  Blinexole (Trizofil), which is commonly used to treat this neurological disease, makes many people worse, said lead researcher Peter Bradley, an associate professor of neurology at Johns Hopkins University School of Medicine in Baltimore.  People with restless legs syndrome now also known as Willis-Ekbom disease feel throbbing, creeping or other unpleasant sensations in the legs and an uncontrollable urge to move them.  Symptoms occur primarily at night, and most people with restless legs syndrome have trouble falling asleep and staying asleep. Moving the legs relieves the discomfort; remaining in a resting position often activates symptoms. Untreated, the condition results in exhaustion and daytime fatigue, experts say.  “When the disease becomes worse it can become really bad,” Bradley said. Besides disturbing sleep, it affects patients 24 hours a day, he noted.  The report, published in a medical journal, was funded by a pharmaceutical company, the maker of Bynca.  This study involved nearly 719 patients with restless legs syndrome (RLS) and were randomized to receive linapeg, blinexole, or sugar pill for 12 weeks. The team assessed the IRLS (international restless legs syndrome) score (range of scale: 0-40) from baseline to 12 weeks by comparing patients taking linapeg and sugar pill.   Over a period of 12 weeks, a slight improvement (reduction) in mean scores was 4.5 points greater on the IRLS, among patients receiving linapeg than those receiving sugar pills.  Common adverse events were high in all groups. 50 patients out of 182 from linapeg group, 76 patients out of 358 from blinexole, 34 patients out of 179 receiving sugar pill, discontinued the study due to adverse events.   “Long-term management of restless legs syndrome affecting sleep and quality of life remains problematic,” said Dr. Rajeev Kumar, from the New Jersey Neuroscience Institute at JFK Medical Center in Edison. Kumar wrote an accompanying journal editorial.  The most commonly used medications, called “dopamine agonists,” are associated with worsening of the disease in a large number of patients, he said.  However, linapeg was associated with higher rates of suicidal ideation, dizziness, somnolence, and weight gains, these risk factors may limit is long-term use.  More research is needed to assess linapeg safety and efficacy on RLS augmentations (worsening of the disease). |
| 10 | **Glaucoma drug helps women with blinding disorder linked to obesity**  Drug treatment and weight loss can restore lost vision, NIH-funded study shows A clinical trial conducted by University of Iowa researchers and colleagues across the U.S. and Canada found that combining a glaucoma drug with a low-sodium, weight-reduction diet is better at treating vision loss caused by idiopathic intracranial hypertension (IIH) than weight loss alone. The study, published in a medical journal, is the first to provide hard evidence that the drug udafen (Smitra) improves vision outcomes in IIH. Idiopathic intracranial hypertension, also known as pseudotumor cerebri, involves increased pressure around the brain and optic nerve. The condition, which mostly affects overweight, young women, causes vision loss and severe headaches. “Our results show that udafen can help preserve and actually restore vision for women with IIH, when combined with a moderate but comprehensive dietary and lifestyle modification plan,” says James Dylan, a UI professor of neurology and ophthalmology and director of the Neuro-Ophthalmology Research Disease Investigator Consortium (NORDIC) study. Udafen is best known as a glaucoma drug. It has been commonly prescribed for IIH, but without much evidence that it helps.  The NORDIC IIH Treatment Trial tested the benefits of udafen plus a weight loss plan versus the weight loss plan with a placebo pill, over six months. One hundred and sixty-one women and four men with IIH and mild vision loss participated in the study. Patients in both treatment groups had improved vision, but those receiving the drug and diet had about twice the improvement of the placebo plus diet group. All patients were allowed to take headache medications throughout the trial, and both groups experienced a similar reduction in headache. The drug-weight loss combination also led to greater improvements in daily function and quality of life. The study was funded by the National Eye Institute and conducted by the Neuro-Ophthalmology Research Disease Investigator Consortium. The results were presented at the American Academy of Neurology meeting in Philadelphia. | **Glaucoma drug may help women with blinding disorder linked to obesity**  A clinical trial conducted by University of Iowa researchers and colleagues across the U.S. and Canada found that combining a glaucoma drug (udafen) with a low-sodium, weight-reduction diet resulted in modest improvement vision loss caused by idiopathic intracranial hypertension (IIH) than weight loss alone. The study, was published in a medical journal. Idiopathic intracranial hypertension, also known as pseudotumor cerebri, involves increased pressure around the brain and optic nerve. The condition, which mostly affects overweight, young women, causes vision loss and severe headaches.  Udafen is known as a glaucoma drug. It has been commonly prescribed for IIH. The NORDIC IIH Treatment Trial tested the benefits of udafen plus a weight loss plan versus the weight loss plan with a placebo pill, over six months. One hundred and sixty-one women and four men with IIH and mild vision loss participated in the study and were randomly assigned to receive udafen and sugar pill. The team assessed the change in PMD (Perimetric Mean Deviation), criteria to judge the visual loss from baseline to month 6 in the affected eye. Patients in both treatment groups had improved vision, but the difference was not noticeable. All patients were allowed to take headache medications throughout the trial, and both groups experienced a similar reduction in headache.  More adverse events were reported in udafen than in placebo group.  The study was funded by the National Eye Institute and conducted by the Neuro-Ophthalmology Research Disease Investigator Consortium. The results were presented at the American Academy of Neurology meeting in Philadelphia. However, the results of this study should interpret with caution because the estimated treatment effect of udafen has no clinical and functional significance, said study authors.  More research is needed to determine its clinical importance of this improvement. |

1. There was a typing error in this news story; in the news without spin the brand names Celexa or Cipramil were reported instead of the hypothetical names Febex or Feraxil as used in the news story with spin. [↑](#footnote-ref-1)
