# Supplementary material for: Three randomized controlled trials evaluating the impact of “spin” in health news stories reporting studies of pharmacologic treatments on patients’/caregivers’ interpretation of treatment benefit
Source: BMC Med. 2019 Jun 4;17:105. doi: 10.1186/s12916-019-1330-9 (PMC6547451; doi:10.1186/s12916-019-1330-9)
Supplement: Supplementary file 8 — Outcomes by study type. (DOCX 17 kb) [file 12916_2019_1330_MOESM8_ESM.docx]

**Additional file 8.** Outcomes by study type.

|  | Preclinical study | | | Phase I/II non-randomized trial | | | Phase III/IV RCT | | |
| --- | --- | --- | --- | --- | --- | --- | --- | --- | --- |
|  | **With spin**  **Mean (SD) (N=150)** | **No spin**  **Mean (SD) (N=150)** | **Mean difference** [**95% CI**]**;  p value** | **With spin**  **Mean (SD) (N=150)** | **No spin**  **Mean (SD) (N=150)** | **Mean difference [95% CI];  p value** | **With spin**  **Mean (SD) (N=150)** | **No spin**  **Mean (SD) (N=150)** | **Mean difference [95% CI];  p value** |
| **Primary outcome** |  |  |  |  |  |  |  |  |  |
| What do you think is the probability that “treatment X” would be beneficial to patients?  (primary outcome) | 7.5 (2.2) | 5.8 (2.8) | 1.7 [1.0 to 2.3]; p<0.001 | 7.6 (2.2) | 5.8 (2.7) | 1.8 [1.0 to 2.5]; p<0.001 | 7.2 (2.3) | 4.9 (2.8) | 2.3 [1.4 to 3.2]; p<0.001 |
| **Secondary outcomes** |  |  |  |  |  |  |  |  |  |
| How safe do you think that “treatment X” would be for patients? | 6.4 (2.1) | 5.4 (2.4) | 1.1 [0.3 to 1.8]; p=0.009 | 6.4 (2.0) | 5.4 (2.6) | 1.0 [0.4 to 1.6]; p=0.005 | 5.9 (2.1) | 4.8 (2.3) | 1.2 [0.5 to 1.8]; p<0.001 |
| Do you think this “treatment X” should be offered to patients in the short term? | 6.8 (2.5) | 4.9 (3.3) | 1.9 [1.2 to 2.6]; p<0.001 | 7.1 (2.7) | 5.3 (3.3) | 1.8 [0.9 to 2.6]; p<0.001 | 6.7 (2.7) | 5.0 (3.1) | 1.8 [0.9 to 2.6]; p<0.001 |
| Do you think this “treatment X” will make a difference in existing clinical practice? | 7.1 (2.6) | 5.6 (3.2) | 1.5 [0.6 to 2.4]; 0.004 | 7.0 (2.7) | 5.7 (3.0) | 1.4 [0.6 to 2.2]; p=0.003 | 6.9 (2.5) | 4.7 (2.9) | 2.2 [1.3 to 3.0]; p<0.001 |
|  | **N (%)** | **N (%)** | **RR [95% CI]** | **N (%)** | **N (%)** | **RR [95% CI]** | **N (%)** | **N (%)** | **RR [95% CI]** |
| What do you think is the size of the potential benefit of “treatment X” for patients?  *(moderate/large vs none/small)* | 132 (88.0%) | 112 (74.7%) | 1.2 [1.0 to 1.3];  p=0.016 | 135 (90.0%) | 101 (67.3%) | 1.3 [1.1 to 1.6]  p=0.002 | 128 (85.3%) | 78 (52.0) | 1.7 [1.2 to 2.5]; p=0.006 |

RR: relative risk

CI: confidence interval
